# Implications of Zoonoses From Hunting and Use of Wildlife in North American Arctic and Boreal Biomes: Pandemic Potential, Monitoring, and Mitigation

**DOI:** 10.3389/fpubh.2021.627654

**Published:** 2021-05-05

**Authors:** Lucy O. Keatts, Martin Robards, Sarah H. Olson, Karsten Hueffer, Stephen J. Insley, Damien O. Joly, Susan Kutz, David S. Lee, Cheryl-Lesley B. Chetkiewicz, Stéphane Lair, Nicholas D. Preston, Mathieu Pruvot, Justina C. Ray, Donald Reid, Jonathan M. Sleeman, Raphaela Stimmelmayr, Craig Stephen, Chris Walzer

**Affiliations:** ^1^Wildlife Conservation Society Health Program, Bronx, NY, United States; ^2^Wildlife Conservation Society, Arctic Beringia Program, Fairbanks, AK, United States; ^3^Department of Veterinary Medicine & Arctic and Northern Studies Program, University of Alaska Fairbanks, Fairbanks, AK, United States; ^4^Wildlife Conservation Society Canada, Toronto, ON, Canada; ^5^Department of Biology, University of Victoria, Victoria, BC, Canada; ^6^Nyati Health Consulting, Nanaimo, BC, Canada; ^7^Department of Ecosystem and Public Health, Faculty of Veterinary Medicine, University of Calgary, Calgary, AB, Canada; ^8^Department of Wildlife and Environment, Nunavut Tunngavik Inc., Ottawa, ON, Canada; ^9^Canadian Wildlife Health Cooperative, Université de Montréal, Montreal, QC, Canada; ^10^Salmon Coast Field Station, Echo Bay, BC, Canada; ^11^United States Geological Survey National Wildlife Health Center, Madison, WI, United States; ^12^North Slope Department of Wildlife Management, Utqiagvik, AK, United States; ^13^Institute of Arctic Biology, University of Alaska Fairbanks, Fairbanks, AK, United States; ^14^University of British Columbia, Vancouver, BC, Canada; ^15^Ross University School of Veterinary Medicine, Basseterre, Saint Kitts and Nevis; ^16^Conservation Medicine Unit, Department of Interdisciplinary Life Sciences, Research Institute of Wildlife Ecology, University of Veterinary Medicine, Vienna, Austria

**Keywords:** wildlife, hunting, zoonotic, pandemic, Arctic, boreal, Indigenous, One Health

## Abstract

The COVID-19 pandemic has re-focused attention on mechanisms that lead to zoonotic disease spillover and spread. Commercial wildlife trade, and associated markets, are recognized mechanisms for zoonotic disease emergence, resulting in a growing global conversation around reducing human disease risks from spillover associated with hunting, trade, and consumption of wild animals. These discussions are especially relevant to people who rely on harvesting wildlife to meet nutritional, and cultural needs, including those in Arctic and boreal regions. Global policies around wildlife use and trade can impact food sovereignty and security, especially of Indigenous Peoples. We reviewed known zoonotic pathogens and current risks of transmission from wildlife (including fish) to humans in North American Arctic and boreal biomes, and evaluated the epidemic and pandemic potential of these zoonoses. We discuss future concerns, and consider monitoring and mitigation measures in these changing socio-ecological systems. While multiple zoonotic pathogens circulate in these systems, risks to humans are mostly limited to individual illness or local community outbreaks. These regions are relatively remote, subject to very cold temperatures, have relatively low wildlife, domestic animal, and pathogen diversity, and in many cases low density, including of humans. Hence, favorable conditions for emergence of novel diseases or major amplification of a spillover event are currently not present. The greatest risk to northern communities from pathogens of pandemic potential is via introduction with humans visiting from other areas. However, Arctic and boreal ecosystems are undergoing rapid changes through climate warming, habitat encroachment, and development; all of which can change host and pathogen relationships, thereby affecting the probability of the emergence of new (and re-emergence of old) zoonoses. Indigenous leadership and engagement in disease monitoring, prevention and response, is vital from the outset, and would increase the success of such efforts, as well as ensure the protection of Indigenous rights as outlined in the United Nations Declaration on the Rights of Indigenous Peoples. Partnering with northern communities and including Indigenous Knowledge Systems would improve the timeliness, and likelihood, of detecting emerging zoonotic risks, and contextualize risk assessments to the unique human-wildlife relationships present in northern biomes.

## Introduction

Emerging infectious diseases (EIDs) are a significant burden on public health and economies, and are increasingly recognized as a global threat (1). EIDs are currently defined by the World Health Organization (WHO) as those that “have newly appeared in a population or have existed but are rapidly increasing in incidence or geographic range (2).” This definition does not clarify between different categories of emergence or re-emergence, nor does it clearly differentiate novel diseases with pandemic potential, such as COVID-19, from those that are variants of old pathogens, new detections of old pathogens with novel technologies, or re-emergence of old pathogens in new regions (2). Thus, the definition does not reflect the very different drivers and significance between diseases and pathogens in terms of global vs. local burden, threat and origin (2). The majority of EIDs (over 60%) are considered zoonotic, with zoonoses defined as “any infection that is naturally transmissible from vertebrate animals to humans” (3–7). There is, however, a need to better differentiate between diseases that originate in animals but are subsequently independently perpetuated in human populations, and those that require an animal host for pathogen persistence, to target research, control, policy and mitigation efforts (2). Infection of people with zoonotic pathogens occurs through contact with infected animals via a variety of mechanisms including: direct contact with bodily fluids (e.g., saliva, blood, urine, feces); indirect contact with surfaces contaminated with an animal's infectious secretions; vector-borne through biting arthropods; foodborne through consumption of contaminated raw or undercooked food; and waterborne, via contaminated drinking water (8). The definition of “risk” usually considers two dimensions: how likely the uncertainty is to occur (probability), and what the effect would be if it happened (impact) (9). Both components are important when establishing the local vs. global risks of zoonoses and emerging diseases of animal-origin, and in considering policy or other interventions.

Severe Acute Respiratory Syndrome (SARS)-CoV-2, considered a novel zoonotic coronavirus, and the causative agent for COVID-19, emerged in December of 2019 in Wuhan, China and rapidly spread globally, to devastating effect. Comparative genomic analysis indicates that SARS-CoV-2 evolved naturally, with bats the likely ancestral reservoir host (10). Given that the animal reservoir for SARS-CoV-2 is yet to be identified, others propose the virus be classified an “emerging infectious disease (EID) of probable animal origin” rather than a zoonosis (2). Many, but not all, early cases of COVID-19 were associated with a market in Wuhan that traded in wildlife (11), and plausible scenarios have been put forth to explain the origin of SARS-CoV-2: evolution in bats or an intermediate animal host before zoonotic spillover to humans in the market or market trade chain (12, 13); or natural evolution in humans following direct zoonotic transfer from bats (12), though purifying selection of SARS-CoV-2 in humans since the start of the pandemic has been weak compared with the significant positive diversifying selection that has occurred in bats since SARS-CoV-2 evolved from its closest known relative RmYN02 (13). SARS-CoV-2 shares some genetic similarity with SARS-CoV, and prevailing evidence suggests that SARS-CoV spilled over into humans via an intermediate host–likely masked palm civets (*Paguma larvata*)–at a wildlife market in southern China in 2002 (14–17). The most devastating pandemics in human history, the Black Death (in the 1300s), Spanish influenza (1918), and HIV/AIDS, all resulted from an initial zoonotic spillover from wildlife (18). Of currently defined emerging zoonoses, over 70% originate in wildlife (6, 19–21) however, very few diseases of wild animal origin persist with ongoing zoonotic transmission, with most human infections from such diseases being acquired through human-human transmission. The frequency of zoonotic disease spillover into humans is increasing (1, 19, 22, 23). This rise has been linked to changing human ecology, due to the growing global human population, and its demand for food, land, and natural resources (5, 19, 20, 24). Escalating anthropogenic activities are increasing contact rates between humans, domestic animals, and wildlife (19, 25–28).

Wild animal consumption-based food systems have been implicated in the emergence of diseases with zoonotic origins (including HIV, SARS, Ebola virus disease, Avian Influenza A), and mounting evidence indicates substantial human health risks from the trade in live wildlife (29–40). Members of the WHO team investigating the origins of the SARS-CoV-2 pandemic recently reported that wildlife farms were the most likely source (41). The trade of wild-harvested meat for food fills a continuum from subsistence-based rural consumption, to extensive commercial trade networks to meet growing urban and international demand for wild animal meat as a luxury product (42). There are calls from scientific, health, conservation organizations, and government officials to end commercial trade in wildlife for human consumption. Closing wild animal markets and the trade in wildlife, is viewed as an expedient measure to reduce the risk of future viral outbreaks, like the COVID-19 pandemic, that threaten human health, well-being, economies, and security at local, regional, and global scales (43, 44). Public health organizations, including the WHO, support the rigorous enforcement of bans on the sale and trade of wildlife for food (45). However, some populations are dependent on wild-harvested food to meet basic nutritional requirements. In the face of disruptions to food supply chains during the COVID-19 pandemic, harvesting and sharing of local foods has helped maintain food and nutrition security for isolated boreal and Arctic communities (46), and harvesting of wildlife, with secondary use of wildlife by-products (i.e., pelts, claws, skulls), forms an important part of traditional economies and socio-economic-cultural well-being of Indigenous Peoples. The World Organization for Animal Health (OIE) considers wildlife all of: (a) wild animals (b) free-ranging feral [domesticated] animals, and (c) non-domestic animals in captivity or farming, though these different categories and interfaces likely pose quite different risks for spillover to humans (2). Infection cycles in densely populated wildlife farms, along trade chains and live markets, are very different to those in natural, free-ranging populations. These crowded and stressful environments are much more likely to facilitate cross-species transmission of pathogens with pandemic potential (2, 32, 47). Based on past experiences, well-meaning initiatives aimed at halting the hunting, trapping, and use of wildlife risk violating the rights of Indigenous Peoples as outlined in the United Nations (UN) Declaration on the Rights of Indigenous Peoples (48), and also threaten the subsistence of non-Indigenous hunter communities.

Indigenous Peoples and local communities across the boreal and Arctic regions (or “northern communities”) have extensive and essential relationships with their environments, including through the harvest and sharing of nutritionally and spiritually important native plants, and wildlife, also known as traditional and country foods (49–53). Limited access to traditional and country foods is a strong predictor of health disparities in Indigenous Peoples across the North, and is correlated to diseases such as diabetes, cardiovascular disease and mental illness (54). Throughout boreal and Arctic ecosystems, subsistence economies support rich and diverse cultures that include the use, sharing, and consumption of wildlife. Subsistence is considered as: the personal consumption of wildlife for food, fuel, shelter, clothing, tools, or transportation; the barter, trade, or sharing of wildlife products in their harvested form with relatives, with others in the local community or with persons in locations other than the local community with whom local residents share familial, social, cultural, or economic ties; and the making and selling of handicrafts from wildlife products, when the wildlife are harvested for the purposes defined above (55). Subsistence, together with guided hunting and fishing, by Indigenous Peoples in the Arctic and boreal is best considered through the lens of food security and sovereignty, cultural security, and livelihoods (56, 57). Food security is considered to exist when “all people, at all times, have physical, social, and economic access to sufficient, safe, and nutritious food to meet their dietary needs, and food preferences, for a healthy and active life” (58). Food sovereignty refers to the “ability and right of people to define their own policies and strategies for sustainable production, distribution, and consumption of food that guarantees the right to food for the entire population” (59). The rights of Indigenous Peoples to determine and maintain these relationships with the environment are embedded in the UNDRIP (48), in the Canadian Constitution (60), and in the Truth and Reconciliation Commission of Canada's Calls to Action (61), but have received less formalized protections within the United States. The relationships between Indigenous Peoples in North America and the environment, particularly the harvest and use of wildlife, are the basis for historic numbered Treaties, and modern land claim agreements in Canada and the United States, Native to Native agreements, and Indigenous advisory and co-management institutions (see Table 1 for specific examples). Such agreements however, have not been as effective in addressing the needs of Indigenous Peoples and local communities as they are forced to adapt to the impacts of biodiversity loss and climate change within historically colonial approaches to environmental management (62). The use of wildlife by non-Indigenous local communities are largely managed through legislation and regulations developed, and enforced, by governments and regional authorities.

**Table 1:** Examples of formal agreements and co-management institutions protecting the rights of Indigenous Peoples in Arctic and boreal biomes across North America regarding land, hunting, and use of wildlife.

**Type of agreement**	**Specific examples of agreements**
Agreements with federal government of Canada	Inuvialuit final agreement; Nunavut land claims agreement; James Bay and Northern Québec agreement and the Northeastern Québec agreement; Nunavik inuit land claims agreement; Eeyou marine region land claims agreement; Labrador Inuit land claims agreement; Umbrella final agreement (Yukon first nations), Historic treaties and agreements^a^ across the boreal, including Treaty No. 5 (Manitoba), Treaty no. 8 (Alberta, Northwest Territories, British Columbia), James Bay treaty–treaty no. 9 and adhesions made in 1929 and 1930 (Ontario), Treaty no. 10 (Saskatchewan)
Agreement with national government of the United States	Alaska native claims settlement act
Indigenous Nation to Nation agreements	Inuvialuit-Inupiat polar bear management agreement (IIA); Alaska and inuvialuit beluga whale committee (AIBWC)
Indigenous advisory and wildlife co-management institutions (not exhaustive)	Alaska: alaska eskimo whaling commission (AEWC); Alaska beluga whale committee (ABWC); Iceseal committee (ICS); Alaska nannut co-management council (ANCC); Eskimo walrus commission (EWC); Indigenous people's council for marine mammals (IPCoMM); Association of traditional marine mammal hunters of chukotka (ATMMHC); Wildlife management advisory council; kitikmeot regional wildlife board Canada: Nunavut wildlife management board, Inuvialuit game council, Fisheries joint management committee, Hunting fishing trapping coordinating committee, Nunavik marine region wildlife board, Eeyou marine region wildlife board, Torngat wildlife & plants co-management board, Sahtu renewable resources board, Gwich'in renewable resources board, Wek'eezhii renewable resources board, wildlife management advisory council (NT, NS), Yukon fish and wildlife management board, makavik corporation

a *https://www.rcaanc-cirnac.gc.ca/eng/1100100028574/1529354437231*.

Indigenous Peoples in the Arctic and boreal are often at the forefront of protecting wild food systems, livelihoods, and cultural values (63, 64), and concerns have been raised about potential impacts to Indigenous rights and food sovereignty due to policy initiatives focused on hunting, consumption, sharing, and local trade of wildlife (55, 65–70). Negative impacts have occurred when socio-economic, cultural, and nutritional dimensions of Indigenous subsistence practices were not considered. For example, previous culturally insensitive and poorly developed communication outreach efforts regarding health and traditional and country foods (on contaminants) resulted in negative health consequences for affected populations from avoidance of traditional foods altogether, given limited healthy alternatives (71, 72). Zoonotic health risks and concerns are also relevant to local communities of hunters, trappers, and fishers across the region. This paper was written to respond to some of the issues related to northern food sovereignty, and to apprehension expressed by researchers and veterinarians working with these northern communities around potential routes for introduction of emerging zoonoses, such as COVID-19, into remote Indigenous and local communities where subsistence based on traditional and mixed economies remains a vital necessity. The manuscript aims to (i) review zoonotic pathogens of wildlife origin in Arctic and boreal systems, in the context of wildlife use by northern communities; (ii) provide a reference for northern communities, and wildlife disease and public health researchers, to consider and compare, the potential community and broader health implications of zoonoses transmissible via traditional use of wildlife; (iii) examine zoonotic pathogen spillover, amplification, epidemic or pandemic spread, and relevant dynamics in northern biomes; (iv) describe some future zoonotic concerns; and (v) discuss considerations for current and future monitoring, surveillance and risk reduction approaches. The results section, with Table 2, addresses aims 1 and 2, and the remaining aims are addressed in the discussion.

**Table 2:** Zoonotic pathogens of Arctic and boreal systems that can be transmitted to humans through hunting, consumption or other use of wildlife, noting pathogen potential for local outbreak clusters, human-to-human transmission, and epidemic/pandemic spread.

**Type of pathogen**	**Disease/Pathogen**	Wild Host(s)^*^ in arctic and boreal biomes	**Route of infection to humans**	**Disease in humans**	**Local outbreak clusters possible?**	**Human to human transmission?**	**Epidemic or pandemic potential?**
Parasites	Anisakidosis/Roundworms of genus *Anisakis* (73, 74)	Definitive host: Bearded seals, ringed seals and beluga whale Intermediate: Fish and squid	Consumption of raw fish	Gastritis with ulcerative lesions of stomach wall	Possible through consumption of shared contaminated product	No	No
	Fluke infection *Cryptocotyle lingua* (fish trematode) (75, 76)	Intermediate host: Fish Reservoir hosts: Fish-eating birds and mammals including foxes, gulls, terns, and herons	Ingestion of raw or improperly cooked fish from fresh and brackish water	Liver and intestinal damage	Possible through consumption of shared contaminated product	No	No
	Cystic Echinococcosis/*Echinococcus canadensis* (22, 77–83)	Definitive host: Wolf, coyote (dog) Intermediate host: Mainly caribou/reindeer and moose (also muskox, elk, bison, and white-tailed and mule deer)	Canids: Ingestion of viscera of infected intermediate host. Humans: Via accidental ingestion of eggs shed in canid feces (e.g., from fur during fox skinning), or from a water or food source (e.g., plants, berries) contaminated with eggs	Relatively benign cyst formation in liver and lung	Possible through contaminated water source	No	No
	Alveolar Echinococcosis/*Echinococcus multilocularis* (79, 84, 85)	Definitive host: Fox, felid, wolf, and coyote (also dog) Intermediate host: rodents e.g., vole, deer mice, lemming, muskrat (ground squirrels and shrews on St. Lawrence Island)	*Canids:* Ingestion of viscera of infected intermediate host. *Humans:* Accidental ingestion of eggs shed in canid or felid feces (e.g., from fur during fox skinning); or from contaminated water or food source (e.g., plants, berries)	Alveolar hydatid disease with parasitic tumor growth in liver, lungs, brain, and other organs and much higher mortality than for *Echinococcus canadensis* infection	Possible through contaminated water source	No	No
	Tapeworms/*Diphyllobothrium latum* & *Diphyllobothrium dendriticum* & *Diphyllobothrium nihonkaiense* (73, 86)	*Diphyllobothrium latum*: Fish-eating mammals (e.g., bear, wolf, otters, and mink) *Diphyllobothrium dendriticum*: Fish, fish-eating mammals, and birds *Diphyllobothrium nihonkaiense*: Wild salmon	Consumption of undercooked fish meat or livers	Asymptomatic or causes mild chronic intermittent diarrhea	Possible through consumption of shared contaminated product	No	No
	Toxocariasis/*Toxocara canis* (primarily) & Toxocara cati^**^ (79, 82, 83, 87–90)	*Toxocara canis:* Wolves, coyotes, and foxes (small mammals), (dog) *Toxocara cati*: Felids (e.g., lynx) and rodents	Mainly through accidental ingestion of eggs from contaminated; possible via consumption of uncooked meat of small mammal paratenic hosts	Ocular and visceral larval migrans	Possible through contaminated water source	No	No
	Toxoplasmosis/*Toxoplasma gondii* (79, 84, 91–101)	Definitive host: Felids (e.g., lynx) Intermediate hosts: many northern animals including caribou, walrus, birds and seal	Consumption of raw or undercooked meat; or via water or soil contaminated with felid feces containing infective oocysts	Often asymptomatic, possible association with mental health issues e.g., depression; severe disease in immune- compromised individuals (e.g., encephalitis/chorioretinitis); fetal morbidity and mortality during pregnancy	Possible through consumption of shared contaminated product	No	No
	Trichinellosis/*Trichinella native* (30, 73, 79, 82, 84, 102–106)	Walrus, seal, bear (polar, black and grizzly), fox, wolf, and wolverine	Ingestion of raw or undercooked meat	From asymptomatic to nausea, diarrhea, vomiting, abdominal pain, muscle pain, fever swelling of eyes, weakness/fatigue, headache, and (rarely) fatality if heart affected	Possible through consumption of shared contaminated product	No	No
	Giardiasis *(Giardia* spp.) & Cryptosporidiosis *(Cryptosporidium* spp.) (107)	Mammals including beaver, muskrats, muskoxen, and others	Via water contaminated with feces containing infective oocysts	Diarrheal disease	Possible through contaminated water source	No	No
Bacteria	Anthrax/*Bacillus anthracis* (108–111)	Wild ungulates (e.g., white-tailed and mule deer, bison, moose, and reindeer)	Ingestion or inhalation, or contamination of wounds by, bacterial spores	Cutaneous: skin sores Inhalational: chest pain, shortness of breath, cough, nausea, vomiting, stomach pains, headache, sweats, fatigue, body aches Gastrointestinal: Fever, swelling of neck glands, sore throat, nausea, vomiting and diarrhea, headache, fainting, swelling of abdomen. All types have potential, if untreated, to spread throughout body, causing severe illness and even death	Possible through shared contaminated water or food source. Rarely direct human-to- human transmission	Very rare reports from cutaneous form. Not considered contagious	No
	Brucellosis/*Brucella* spp. (81, 84, 112, 113)	Wild mammals including: caribou/reindeer, elk, muskoxen, bison, white-tailed and mule deer, goats, sheep, moose, wolf, fox, rodents, hares, mink, and marine mammals	Handling of carcasses, fetuses, and newborn calves from infective animals; or consumption of raw (including frozen or dried) meat and marrow Dogs: Consumption of uncooked infected tissue	Systemic bacterial disease (acute or insidious): intermittent fever with headache, weakness, sweating, chills, joint pain and weight loss; also cerebral forms; can be fatal	Possible, through consumption of shared contaminated product	Extremely rare (e.g., through breastmilk)	No
	Botulism/Clostridium botulinum^***^ (114–119)	Fish (especially salmon) and many mammals Main source in north is marine mammals, especially seals and whales	Consumption of raw or parboiled seal meat, fish, seal oil, or other wild meat that has undergone faulty fermentation or aging	Multiple clinical symptoms including: blurred vision, nausea, vomiting, paralysis of the motor nerves, and respiratory paralysis in fatal cases	Possible, through consumption of shared contaminated product	No	No
	Erysipelas/*Erisipelothrix rhusiopathiae* (56, 120–128)	Terrestrial and aquatic mammals including muskoxen, white-tailed and mule deer, caribou, birds, fish, and arthropods	Exposure to infected animals or fish or animal products via skin wounds or via ingestion; environmental sources of infection also reported	Localized skin infections; or severe cases with diffuse cutaneous or systemic disease, septicemia, endocarditis; infrequently pneumonia, abscesses, meningitis, arthritis	Unlikely	No	No
	Leptospirosis/*Leptospira interrogans* (129–131)	Beavers, coyotes, white-tailed and mule deer, foxes, opossums, otters, raccoons, skunks, and Northern fur seals	Direct contact with contaminated urine or animals	From no symptoms, to kidney damage, meningitis, liver failure, respiratory distress, and death	Possible through contaminated water source	No	No
	Lyme disease/*Borrelia burgdorferi* (132, 133)	Seabirds, song birds, and wild ungulates	Bites from ticks that have fed on an infected animal	Fever, rash, facial paralysis, arthritis	Possible if infected host and tick densities high	No	No
	Pasteurellosis/*Pasteurella multocida; Bisgaardia hudsonensis* (others) (134–137)	Pinnipeds, including seals and walruses, many terrestrial mammals, birds, and reptiles	Animal bites or contact with nasal secretions of infected animal	Skin and soft tissue infections: rapidly spreading edema, erythema and tenderness at site of the bite or scratch; abscessation; enlarged local lymph nodes	No	No	No
	Q fever/*Coxiella burnetii* (138–140)	Northern fur seals and sea birds	Inhalation of dust contaminated by infected animal feces, urine, milk, or birth products; contaminated water source; or ingestion of infected animal products e.g., milk or cheese	Mild: Fever, fatigue, headache, muscle aches, vomiting, diarrhea, chest or stomach pain, weight loss, cough Severe: pneumonia or hepatitis. Infection during pregnancy can cause miscarriage, stillbirth, pre-term delivery, low infant birth weight	Occasionally, through shared contaminated water or food source, or human-to-human transmission	Rare: although highly transmissible from animal-human it is not highly transmissible from human-human	No
	Seal finger/*Mycoplasma* spp. (114, 134, 141)	Seals and whales	Marine mammal bites; or broken skin contact with infectious material from marine mammal	Swollen, painful, and suppurative lesion on finger; rarely systemic, with fever and lymphangitis	No	No	No
	Tuberculosis and Mycobacteriosis/*Mycobacterium bovis, M. tuberculosis, M. pinnipedii* & others (114, 142–144)	Marine mammals: Wild seals (more commonly) and cetaceans (rarely) Terrestrial mammals: bison, elk, moose, white-tailed deer, mule deer and wolves	Multiple routes: inhalation, ingestion of raw/undercooked meat or unpasteurized milk products, and direct contact with breaks in the skin e.g., when dressing infected ungulates	Pulmonary (cough, shortness of breath) and cutaneous (localized skin infections) disease	Possible, through consumption of shared contaminated product and direct human- to-human transmission	Yes	Yes
	Tularemia/*Francisella tularensis* (84, 145–148)	Muskrats, beavers, hares, voles, squirrels, wolves, bears, and other northern wildlife	Consumption of insufficiently cooked meat or contaminated water and dust; or through bites from infected vectors such as mosquitoes and ticks; and through direct contact i.e., skinning; touching hare carcasses	Skin lesions or ulcerations, lymphadenomegaly, vomiting, diarrhea, abdominal pain, conjunctivitis, pneumonia, septicemia, and hepatosplenomegaly	Rare, but possible through consumption of shared contaminated product or contaminated water source or vector abundance	No	No
	Yersiniosis^****^/*Yersinia pseudotuberculosis* & *Y. enterocolitica* (94, 142, 149–151)	Reservoirs in rodents (beaver, muskrat, ground squirrels), lagomorphs (snowshoe hare), and outbreaks in muskoxen	Ingestion of raw or undercooked meat, or water contaminated with infected fecal matter	Fever, abdominal pain, and diarrhea	Possible through consumption of shared contaminated product or contaminated water source	Very rarely; not highly transmissible	
Viruses	Avian influenza/Influenza A viruses (114, 152–155)	Wild birds, especially waterfowl	During preparation of infected birds for eating (plucking, cleaning, butchering) or consumption of raw meat from infected bird	Mild to severe illness, sometimes death. Fever, chills, cough, sore throat, congestion, body aches, headache, fatigue; vomiting, and diarrhea in children	Yes	Yes	Yes
	Caliciviruses/*(marine caliciviruses:* serotypes of vesicular exanthema of swine virus*)* (114, 156)	Arctic marine mammals, including fur seals, elephant seals, walrus, and whales (including bowhead and gray)	Broken skin contact with infectious animal or their secretions	Fluid-filled blisters on the extremities	Rare, but possible through handling same animal	Possible but rare, through broken skin contact with blister fluid	Not for marine serotypes
	Sealpox/*Parapox virus* (114, 157, 158)	Harbor and gray seals	Direct contact via pox lesions on infected mammals	Painful, nodular lesions	Rare, but possible through handling same animal	No	No
	Orf/*Parapox virus* (157–159)	Muskoxen, mountain goats, Dall's sheep, caribou and white-tailed, and mule deer	Direct contact via pox lesions on infected mammals	Painful, nodular lesions	Rare but possible through handling same animal or if multiple animals infected in herd	No	No
	Rabies (38, 81, 160–170)	Principle reservoir hosts: Arctic foxes, red foxes, wolves, and bats Less commonly: Caribou, beaver, black and polar bears, racoons, lynx, and wolverine)	Humans and domestic dogs: via bites from infected wildlife	Almost always fatal if untreated. Affects central nervous system: general weakness or discomfort, fever, headache; prickling sensation at site of the bite, anxiety, confusion, agitation, delirium, hallucinations, hydrophobia (fear of water), and insomnia	Possible through rabid animal in community	Extremely rare: through bite or organ transplant	No
	Hepatitis E^*****^ N.B. transmission from wildlife to humans not yet confirmed in Arctic or boreal regions, but suspected (171–173)	Free-ranging deer, possibly caribou	Humans via undercooked meat or food contaminated with feces from infected animal	Acute viral hepatitis, mortality a concern in pregnant women	Possible through consumption of shared contaminated undercooked meat or shared contaminated water source	Rare: mainly via maternal-infant transmission	Outbreaks possible via fecal contamination of drinking water source
Fungal	None as yet	Future concern in warming Arctic and boreal systems. Dearth of data for fish (174)	Future concern in warming Arctic and boreal systems	N/A	N/A	N/A	No
Prion Diseases	Chronic Wasting Disease (CWD)^******^ (175, 176)	Wild cervids: Moose, white-tailed and mule deer, elk and reindeer in Fennoscandia (potentially caribou)	No documented transmission to humans as yet, but experimental evidence of CWD transmission to non-human primates. Other TSEs have spread from animals to humans via consumption of infected offal, so public health officials still advise caution	N/A	Potentially possible through consumption of shared contaminated product	N/A	No

* *Definitive or final host = host organism in which a parasite reaches maturity (adult stage) and reproduces sexually; Intermediate host = host organism that harbors the sexually immature parasite and is required by the parasite to undergo development and complete its life cycle; Reservoir host: primary host that maintains a pathogen in a system and that serves as a reservoir of infection for other species*.

** *Toxocara cati is rarely zoonotic*.

*** *Not strictly a zoonosis, but of concern in Indigenous communities and contracted via raw/fermented wild meat*.

**** *Yersinia pestis discussed in **Appendix 1** as a future concern; not yet reported from humans in the Arctic*.

***** *There is currently no confirmed transmission of Hepatitis E virus from wildlife to humans in the Arctic and boreal regions, however one study found serological evidence of HEV infection in 3% of the observed Canadian Inuit population (171)*.

****** *As yet, there has been no documented transmission of CWD to humans, but caution is still advised: other Prion diseases have spread from animals to humans e.g. Bovine Spongiform Encephalopathy/Mad Cow Disease, and have a very prolonged incubation period in people; experimental research has shown potential for transmission to non-human primates and ability of CWD to convert human prion protein to a misfolded state; CWD is an emerging disease and more longitudinal research is warranted (176)*.

## Materials and Methods

We carried out a qualitative literature review to identify publications focused on zoonotic diseases in Arctic and boreal biomes with a potential wildlife origin. The review focused on zoonoses associated with the hunting, trapping, butchering, sharing, use, and trade of wildlife (including fish), and only considered agricultural or domestic species if related to a sylvatic cycle. Given this focus, vector-borne zoonoses were not included in the review, but are considered in the discussion. In addition, we asked for the contributions of experts on the topics. Only published materials were included. Our search included articles, reviews, proceedings papers, reports and book chapters in the English language. The Web of Science database and Google Scholar search engine were utilized to conduct the search in late April, 2020. Publications from any year were considered.

To gain an overview of the literature pertaining to existing zoonotic disease reports for communities in Arctic and boreal regions associated with consumption and/or hunting of wildlife, search terms were conducted using the following keywords and phrases:

➢ “zoonoses” OR “zoonotic”
➢ AND “wildlife” OR “fish” OR “hunter” OR “hunting” OR “hunted” OR “subsistence” OR “country food” OR “traditional food” OR “Indigenous”
➢ AND “Arctic” OR “boreal” OR “Alaska” OR “northern Canada”

Based on the titles and abstracts of the identified studies, we excluded those publications considered irrelevant to part one of our review, including those for which the full manuscript was not available, publications pertaining to vector-borne zoonoses in Northern regions; those describing zoonoses in other regions, or for which hunting, consumption, use or sharing of wildlife have not been reported to facilitate transmission; and those referring to experimental rather than natural infections. Relevance was attributed if the publications discussed case studies of zoonotic diseases transmitted to humans via the hunting, consumption, preparation or sharing of wildlife in northern regions of North America; or if they described the ecology and epidemiology of zoonoses with one or more wild hosts and potential transmission to humans through the hunting, consumption, preparation or sharing of wildlife in North American Arctic and boreal biomes; details on these relevant aspects were recorded in a table.

Ninety five publications were identified for inclusion in the review. The lead author read each full article and extracted relevant information for inclusion in the manuscript. The other authors reviewed this selection and recommended additional papers, where available, that met inclusion criteria described above. All authors then considered together how the review findings combine with risk factors for zoonotic disease emergence, amplification and pandemic spread, and key cultural, socio-economic, political and ecological factors in Arctic and boreal regions, to inform on the risks of zoonotic disease emergence and amplification from Northern wildlife use and trade, and how such risks are being and could be better addressed.

## Results

Our review identified 25 zoonotic bacterial (*n* = 12), parasitic (*n* = 9), and viral (*n* = 4) diseases described across Arctic and boreal regions of Canada and Alaska that can be transmitted to humans through the hunting, consumption, preparation, or other use of wildlife (22, 56, 73, 75–84, 86–95, 97, 99, 102–110, 112–117, 120–125, 129–134, 138–142, 145, 149–154, 157–170, 177–193). Table 2 summarizes the diseases, their causative agents, wild hosts, modes of transmission, and potential for local, epidemic or pandemic spread, with additional details given in the **Appendix 1**. Potential wild species hosts of these 25 zoonoses include many subsistence species important to northern Indigenous Peoples and local communities. These come from various taxa, including carnivores, ungulates, rodents, birds, marine mammals, and fish. In contrast to the global overrepresentation of viruses as emerging and pandemic human pathogens (21, 194–196), viruses comprise the lowest proportion of northern zoonoses. Of the 25 zoonotic diseases reported, 12 were identified as having potential to cause local outbreak clusters through the sharing of contaminated wild animal meat, or other products for consumption (anisakidosis, trematodosis, tapeworms, toxoplasmosis, trichinellosis, anthrax, botulism, brucellosis, mycobacteriosis including tuberculosis, Q fever, tularemia, yersiniosis); 8 have potential for human to human transmission, [anthrax, brucellosis (via breastmilk), influenzas, Q fever, tuberculosis/mycobacterial disease, yersiniosis, caliciviruses, and rabies]; and only 2 of these [influenzas and tuberculosis/mycobacterial disease] are considered as having true epidemic or pandemic potential. Many of these pathogens can threaten human health directly as zoonoses, but also indirectly via food and economic insecurity, by causing mortality events and declines in wildlife, or reduced quality of wildlife products on which northern Indigenous Peoples and local communities depend (120, 178, 179, 193). Thus, these zoonotic diseases are of local significance, even where EID risk of more global significance is low.

A study of 36 Inuit communities for 4 zoonotic parasites, found highest levels of exposure to *Toxoplasma gondii* (27.2%) and trichinella (18.6%), with overall seropositivity related to age, education, and consumption of marine mammals and seafood (180). Serological surveys of hunters and trappers in Cree communities in northern Québec (Eastmain, Wemindji, and Mistissini) found 44% to be positive for at least one zoonotic pathogen, with risk correlated to hunting, fishing, and trapping activities, as well as consumption of smoked game, and domestic dog ownership (91, 181). Free-ranging domestic dogs serve not only as sentinels for disease, but also as a conduit for diseases from wildlife to people e.g., for rabies and zoonotic parasites which dogs acquire from wild meat (78, 87, 182–184). The role of dogs as potential sources or amplifying hosts for zoonotic diseases highlights the importance of integrating improved domestic animal management and healthcare alongside that for humans in northern communities (197).

While wildlife use in Arctic and boreal biomes is not currently considered a major concern in terms of being the source of an epidemic or pandemic, zoonotic diseases of wildlife remain important for northern communities in the Arctic and boreal biomes: changes in quality and quantity of wildlife, access to traditional and country foods, and potential for disease risk, can create public health, food safety, and food security concerns. Indigenous and local communities in northern and remote areas of these biomes also tend to have limited access to health care services, along with suboptimal housing, infrastructure such as potable water, and sewage treatment facilities. These underlying factors contribute to the cumulative impact of additional stressors like zoonoses on the community (198).

## Discussion

### Zoonotic Pathogen Spillover, Amplification, Epidemic or Pandemic Spread, and Relevant Dynamics in Northern Biomes

Spillover and amplification of novel diseases is a rare, dynamic, and complex process with multiple factors at play (25, 47, 199–203). Zoonotic disease emergence events can result in dead-end “spillover” infections in which the pathogen is unable to establish stable onward transmission in the novel (human) host (27, 204). Despite this, spillover events are thought to be under-reported, as they can occur in remote regions where people have limited access to healthcare and reporting, with a lack of surveillance and diagnostic test services, and limited infrastructure, while also living in relative isolation from other human populations (47). Novel diseases are likely often mis-diagnosed, or not diagnosed at all in mild cases that resolve without treatment (47). The risk of pathogen spillover from reservoir hosts to humans, or other animals, depends upon the intensity of infection within reservoir host populations, and human contact with the reservoir population, and is affected by a cascade of events involving multiple factors associated with hosts (reservoir, intermediate/vector, recipient), the environment and the pathogen itself (205–208). This includes release of infectious material from reservoir hosts, pathogen survival in the environment, behaviors that drive exposure of a novel host, and biologically driven susceptibility of that host (199, 209). For a spillover event to result in epidemic or pandemic spread, it may need to overcome multiple barriers (210), and the pathogen must adapt to efficiently spread between individuals of the recipient population, with genetic, physiological and immunological attributes of the recipient host, together with dose and route of exposure, affecting the susceptibility to infection (206, 211). The pathogen can then be considered as transformed from a zoonotic EID to a pathogen specific for the recipient population (2). High densities, and gregariousness/sociability of the recipient host species can then facilitate pathogen exposure and transmission. Similarly, connectivity through travel, and trade networks facilitate regional, international, and global pandemic spread of pathogens in humans and domestic animals (212, 213).

Of human pathogens with potential for widespread transmission and global dissemination i.e., pandemic potential, a disproportionate number are viruses (20, 21, 26, 194–196, 214). Viruses transmitted to humans during practices that facilitate mixing of diverse animal species have significantly higher host plasticity, and higher pandemic potential (201). Zoonotic viruses capable of infecting a more diverse range of host species have higher pandemic potential in humans than those with a narrower host specificity: they are more likely to be amplified by human-to-human transmission, and spread on a global scale (201, 215). Host traits play a role in transmission, and the proportion of zoonotic viruses per mammal species has been linked with phylogenetic relatedness to humans, host taxonomy, host biomass and density, and opportunities for human contact (194, 211, 216–219). Certain mammalian groups (bats, rodents, primates) have been suggested as more likely to host zoonotic viruses (194, 215); though recent analysis suggest bats and rodents host high numbers of zoonoses simply due to the high degree of species richness within these orders, not as a result of intrinsic or ecological differences (220). Whilst wildlife are described as the source of the majority of emerging zoonotic diseases, the term “wildlife” is defined by OIE as “all free-roaming wild animals, feral animals and captive or farmed wildlife” (2). It is valuable to differentiate spillover risk posed by free-roaming wildlife, vs. captive-managed “farmed” or traded wildlife that may have wild origins, but have either been caught and maintained, or bred in captivity. Wildlife trade chains and markets that trade and process live animals or fresh meat from wildlife frequently represent high-risk interfaces due to high wildlife-human contact rates, and large numbers of admixed species (including mixing of wild and domestic animals), with their potential to shed and share viruses for extended periods prior to on-site slaughter or onward sale (221, 222). The proportion of wild rodents testing positive for coronaviruses has been shown to significantly increase along the live animal supply chain from local traders (21%), to large markets (32%), and to restaurants (56%) (32). Environmental and physiological stressors, such as the poor transportation and holding conditions along wildlife trade chains, impact animal immune function, and can lead to increased shedding and amplification of viruses (210, 223–225).

The risk of spillover of zoonotic diseases from wildlife increases when wildlife-human, and wildlife-domestic animal-human contact rates increase (19–21, 25, 42). Ecosystem degradation, land-use change for agricultural intensification or other industrial expansion, wildlife trade, and wildlife markets, all contribute to increasing contact rates, and therefore increase risk of disease emergence, particularly when they occur in areas of high biodiversity (19–21, 25, 47, 226–241). A higher diversity of zoonotic agents is found where the diversity of host animals is higher (239, 242). Although higher animal host and pathogen species diversity is associated with an increased risk of disease emergence (243), complex host, environmental and pathogen relationships ultimately determine spillover risk (206, 244). A high level of biodiversity can serve as a source of pathogens, but can also serve as a regulating factor (termed the “dilution” effect), where by loss of habitat specialists and predators due to habitat disturbance or hunting, can allow more generalist reservoir host species, such as some rodent species, to proliferate (240, 245). Zoonotic viral richness correlates strongly with mammalian species diversity and abundance (2), and domestic species harbor, on average, 19.3 zoonotic viruses compared to an average for free-ranging wildlife of 0.23 (2). Wildlife make up <1% of the world's non-human mammal biomass, thus, whilst wildlife is of concern as a source of uncommon but significant animal-origin EIDs, land-use change for agricultural intensification to supply human food systems, particularly for livestock, is of concern as a significant driver of EID risk (19, 24, 238). Higher biodiversity tends to be observed along ecosystem edges (e.g., along roads built into pristine ecosystems, or fragmented forest edges) where differing ecological systems meet, resulting in increased contact opportunities between a wider range of different host and pathogen species, and increased potential pathways for spillover (237, 246, 247). In biodiverse areas, livestock can become intermediate or amplifier hosts in which pathogens can evolve and spill over into humans (238) and over three-quarters of livestock pathogens are capable of infecting multiple host species (219).

### Wildlife Use, Spillover and Emerging Zoonotic Diseases in North American Arctic and Boreal Regions: Social, Cultural, Political, and Ecological Considerations

**Figure 1:**
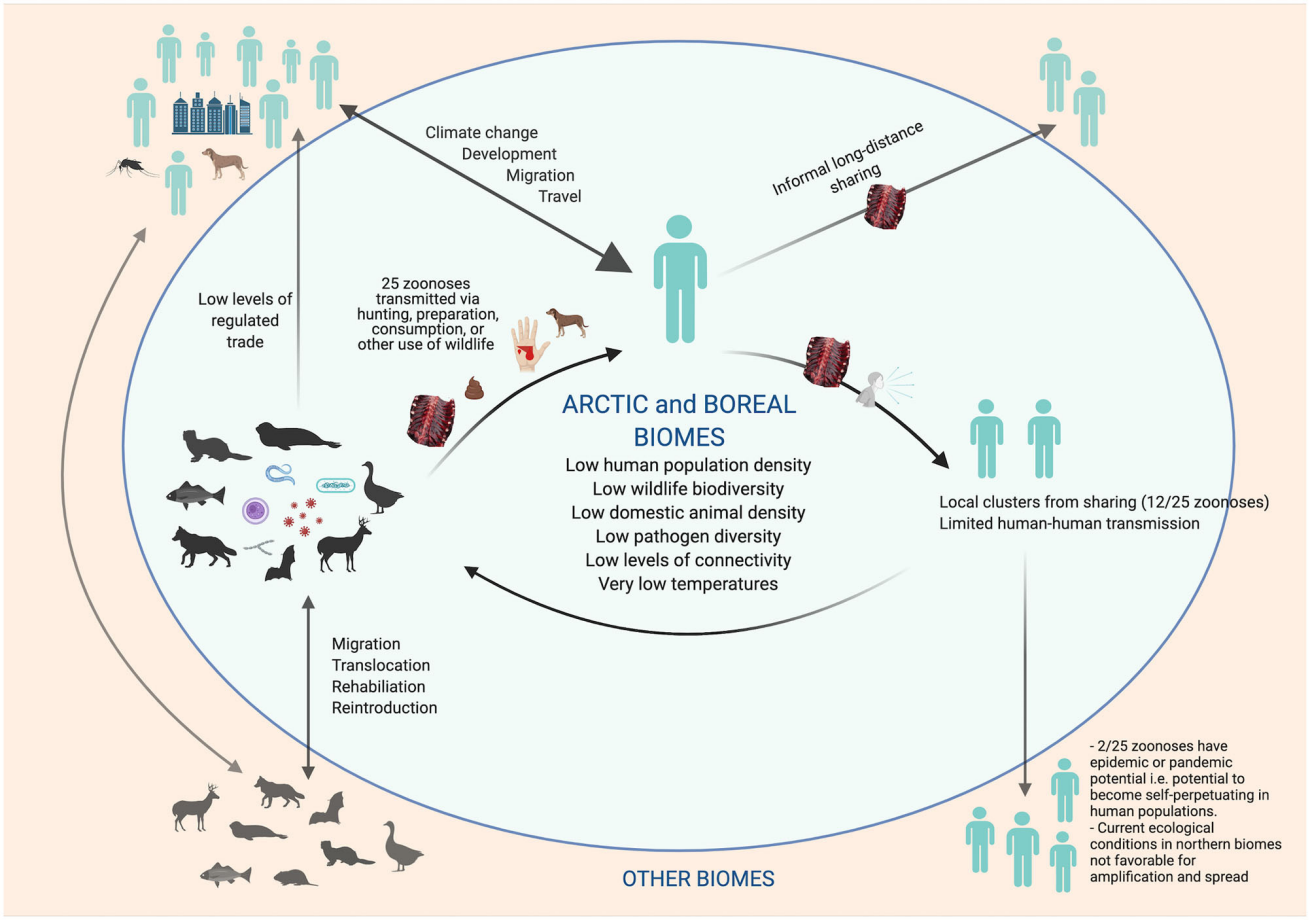
Transmission of Zoonoses of Wildlife Origin in Arctic and Boreal Regions: Characteristics That Lower Risk for Novel Pathogen Emergence and Pandemic Spread (Created with BioRender.com).

Arctic and boreal socio-ecological systems are currently less predisposed for novel zoonotic disease emergence, due in part to a relatively low species diversity; low population density of human and livestock; limited and less intensive land uses, such as for commercial forestry and agriculture; and a lack of live wildlife markets, which are not a part of the cultural values of northern communities (19, 25, 206, 220, 239, 248–252). In addition, cold temperatures, photoperiod extremes, and geographic isolation restrict the diversity of pathogens, and often require specialized pathogen adaptations in order to persist in these environments (250, 253) (Figure 1). Recent mapping of global hotspots of relative risk for zoonotic EID events based on demographic, environmental, and biological correlates found Arctic and boreal biomes to be within the lowest risk index (25). Low human density, and reduced connectivity among human populations, also decrease the likelihood of onward pandemic spread of any newly emerged pathogen (28, 213).

Commercialization of wildlife for food has been, and continues to be promoted in northern communities and regions. The type, and context, of sharing and trade of wildlife for food in northern communities is entirely different in character from the commercial trade in wildlife for human consumption, including wildlife markets, in parts of Asia and Africa. The trade is also not characterized by the same mixing of species carrying potential pandemic zoonotic pathogens as commonly occurs in wildlife trade chains throughout tropical regions. As an example, a Nunavut Wildlife Harvest Study found only 86 records (from over 145,000, and after removing the separately-monitored commercial harvest of muskoxen (*Ovibos moschatus*) and sales to fish or meat plants) indicating a harvest was sold commercially, vs. for personal use (254). Larger-scale commercial trade of wildlife from Arctic and boreal biomes is highly regulated, and falls under similar food safety inspection as meat from domestic animals, which not only lowers the risk of zoonotic disease transmission, but also protects the food and economic security of Indigenous and local communities who rely on wildlife (255–257). Whilst commercial trade still forms only a small component of wildlife use, the dual roles of wildlife harvesting in northern subsistence and mixed economies, including sport hunting, remain important (258–260). Maintaining and strengthening current surveillance systems for wildlife health and zoonoses, based on populations with the highest contact rates with wildlife, is an important component of an improved health infrastructure across the North. These systems can help detect, define, and control local human emergence of zoonoses while still geographically confined.

Risk for emergence of zoonotic-origin pandemic pathogens from wildlife use in Arctic and boreal biomes is currently minimal, indicating that new policies restricting these traditional and subsistence activities in the name of pandemic prevention would be unfounded at the current time.

Our review did find that endemic zoonotic concerns persist in northern communities (Table 2, **Appendix 1**), and increased risk is associated with consumption of raw meat, the practice of meat and fish fermentation (e.g., *igunaq, muktuk*), and exposure to the bodily fluids of animals through methods of harvesting and butchering (56, 118). For many Indigenous cultures around the Arctic, wildlife as food, and food sharing, are fundamental components of a cultural value system that emphasizes generosity, reciprocity, and cooperation, and usually operates within networks related to kinship and family social groups within the community (261–267). Alaska prohibits the sale of most wildlife hunted by Alaska Natives to non-Alaska Natives, but, across Canada, different jurisdictions recognize different Indigenous rights and responsibilities with respect to disposition of country food. For example, in Nunavut, an Inuk has the right to dispose freely to any person any wildlife lawfully harvested (268). Food exchanges are important because of the nutritional value of the items exchanged, and also because they carry cultural and economic values (53, 269, 270). Local sharing of foods can facilitate community zoonotic disease outbreaks (e.g., trichinella or botulism, as noted in Table 2); however, the limited potential for human-to-human transmission of current pathogens spread via sharing, and the low human population density, makes the likelihood of epidemics and pandemics originating from such activities very low. While this traditional way of sharing remains, there also exist other broader networks of sharing practices established to support urban hubs such as Anchorage, and others, across the United States (265, 266). Although low levels of consumption and sharing of traditional foods by Indigenous Peoples in urban centers in Canada have been previously reported (64, 271), relatively new sharing networks have emerged as a result of social media (272, 273) that facilitate the broader distribution of country food, often at the expense of local and traditional sharing practices and values (274), and potentially facilitating the spread of pathogens across larger geographic regions. Zoonotic disease risk is lowered through tight regulation of legal trade of wildlife and wildlife products from northern regions, however international spread of a zoonosis has been associated with sport hunting and the illegal export of wild meat (275).

Despite the perceived current low risk for zoonotic EID events, Arctic and boreal biomes are undergoing rapid ecological change. As the earth warms, and permafrost thaws, concerns have been raised for the potential release of dormant pathogens and contaminants that could affect wildlife and humans. Climate change, migration, introduction of industrial land-use, alongside increasing connectivity of people through development of tourism, travel and trade networks, will alter pathogen dynamics and could facilitate the future spread of emerging pathogens both to and from Arctic and boreal biomes (28, 213, 276, 277). These trends are already reflected in range expansion of existing pathogens (278, 279), and could promote reemergence of past diseases, novel host-pathogen relationships, and the emergence of new zoonotic diseases including vector-borne diseases (121, 185, 277, 280–282). Novel pathogens have recently emerged in northern regions: A second case of Alaskapox virus was reported in Fairbanks in October, 2020, with suspected, but as yet unknown, zoonotic origin (283), and Erysipelothrix has emerged as an apparently new disease causing agent in muskoxen in Canada, with a genotype distinct from strains found in other regions (121).

The greatest risk for pathogens of pandemic potential being introduced into northern communities is currently from people coming into the region from more densely populated southern areas (e.g., via cruise ship passengers). Travel and tourism were found to be the most significant and frequent drivers of epidemic events in Europe, and during the ongoing COVID-19 pandemic (284, 285). The current COVID-19 outbreak is an excellent example of travel-related introduction into northern regions, and subsequent community spread of a suspected wild-origin EID maintained by human-human transmission (286). As a result, many remote northern communities, who experienced impacts from previous epidemics such as influenzas, smallpox, and tuberculosis (287), have imposed strict travel restrictions and prohibited outsiders from entering their communities during the current pandemic (288). Northern residents traveling to and from regional hubs for medical procedures and other essential activities should be considered in future risk scenario planning, particularly during a pandemic. Severe Acute Respiratory Syndrome (SARS) like coronaviruses have not been identified in any wild animals in Arctic and boreal biomes. Natural and experimental infection indicates that SARS-CoV-2 appears to have the ability to infect a broad range of distantly related mammals (289). Analysis of Angiotensin-converting enzyme 2 (ACE2) receptors, suggests that some cetacean species are hypothetically susceptible, including beluga (*Delphinapterus leucas*) and narwhal (*Monodon monoceros*) (261) which are hunted by northern communities. However, entry of a virus into a cell is a complex biochemical process, with multiple other factors at play beyond the binding to a receptor, and actual infection of marine mammals is undocumented. Marine mammals are not deemed a risk based on current knowledge. Other coronaviruses have previously been identified in marine mammals, but there has been no evidence of zoonotic transmission from these animals to humans (290–293).

Reverse zoonotic transmission of human pathogens to non-human animals (anthropozoonoses), including wildlife, occurs more frequently than previously thought (294), and this directional flow of infection for SARS-CoV-2 from humans to wildlife is currently more likely than the reverse in Arctic and boreal biomes. A recent assessment found a non-negligible risk of transmission of SARS-CoV-2 from humans to bats (295), and, whilst the COVID-19 pandemic is ongoing, suspension of field work that involves direct interaction with bats has been recommended (296). Preventing human-to-wildlife SARS-CoV-2 transmission is important for protecting animals from disease, but also to avoid establishment of reservoirs in wild animals (e.g., bats), complicating disease control efforts, with potential for future spillover to other wildlife (e.g., Mustelidae) and spillback to humans (297, 298). Both anthropozoonotic, and zoonotic transmission of SARS-CoV-2 has been reported on mink (*Mustela vison*) farms across Europe, and the United States (299, 300). Mustelids are highly susceptible to SARS-CoV-2, an important consideration given that trapping of marten (*Martes americana*) and wolverine (*Gulo gulo*) forms a critically important industry in many northern communities (301). Contaminated wastewater and refuse from human settlements, commercial vessels, and cruise ships could theoretically pose a risk of transmission of SARS-CoV-2 from humans to marine mammals in the Arctic (261, 302). However, it is unclear if the virus remains viable under these varying environmental conditions and risk likely remains low (303), and is further attenuated by the current ban on cruise ships in the Artic.

### Future Zoonotic Disease Concerns

The circumpolar North is uniquely vulnerable to the health impacts of climate change, including, but not limited to, alterations in the distribution and ecology of infectious diseases, expansion of zoonotic disease vectors, changing migration patterns, impacts on food security, limited resources of northern communities to respond to medical emergencies, and changes in water availability and quality (65, 227).

Climate change can impact distribution, life cycle, and physiological status of hosts, pathogens, and vectors, and can drive novel cross-species viral transmission (276, 304, 305). For example, a marked increase in leptospirosis was observed in Ontario following the warmest and third wettest autumn in a decade (186). Leptospirosis has not yet been recorded in humans in the Arctic, but serosurveys of Alaskan wildlife found antibodies to *Leptospira* serovars in caribou (*Rangifer tarandus*), moose (*Alces alc*es), and bears (Ursidae) (186). The cumulative impacts of environmental and climatic changes may be increasing the susceptibility to the bacteria *Erysipelothrix rhusiopathiae* in certain wild animal host populations, including multiple unusual mortality events of muskoxen in Nunavut, the Northwest Territories, and Alaska, and of moose and caribou in British Columbia (120–122). In addition to the health risk for people who interact with these animals or their environments, there is potential for reduced food security for northern communities, through the direct loss of animals from *E. rhusiopathiae* (56, 120, 123). Climate change is already and will lead to further changes in wildlife community dynamics, including range shifts and increasing overlap between marine and terrestrial ecosystems as a result of sea ice loss and other climate-related changes. *Yersinia pestis*, a bacterium that persists in rodent-flea communities and causes plague in humans, is not yet reported in the Arctic, though it has been identified twice from wildlife in Canada [in bushy-tailed woodrats (*Neotoma cinerea*) and a Prairie dog (genus *Cynomys*)] (149), and appears to occur enzootically in southern Alberta, Saskatchewan, and British Columbia (150). If warming leads to northern expansion of rodent reservoirs, the disease could affect Arctic and more boreal communities in the future. Northward expansion in mammal ranges are already noted, for example in beavers (*Castor canadensis*) increasingly colonizing the north. Such expansion impacts zoonotic disease ecology, with beavers, for e.g., capable of carrying tularemia, and amplifying and maintaining *Giardia* (249). Migratory terrestrial and marine intermediate hosts have been implicated in the introduction of other zoonoses to northern regions. For example, human seroprevalence for the protozoan parasite *Toxoplasma gondii* is high in some parts of the Canadian Arctic, with infection associated with consumption of under-cooked country foods (306). The parasite, thought originally to be of South American origin, but now ubiquitous around the globe, has felids as the only known definitive host. However, seropositive wildlife species have been detected in the Arctic, where wild felids do not occur, and hypotheses for arrival in the region include spread via migratory waterfowl or marine mammal intermediate hosts (306, 307). As environmental conditions change, bacterial infections routinely reported from direct contact with, or ingestion of, fish in more southern regions may also become a greater concern for northern communities who depend on fish for subsistence and as part of their food sovereignty (126). While there are currently no known zoonotic viruses of fish origin, there are many viruses among fish that share evolutionary history with modern human viral pathogens, and viruses have been shown to readily jump between species in the aquatic environment (308). The possibility of some diseases decreasing in relevance warrants consideration when assessing future potential risks from climate change (187, 253, 309).

Thawing of permafrost has already contributed to changing patterns of traditional food consumption, and forced some northern communities to abandon the use of traditional ice cellars and increasingly utilize preservation methods such as smoking, pickling, and salting, and other ways to store traditional and country foods (281).

If pathogens emerge from thawing permafrost, ice patches, glaciers, or graves, wildlife may be the first to be affected. Wildlife may be both important sentinels and amplifying hosts, and a food safety concern (281). In 2016 in the Russian Arctic, one fatality from anthrax occurred among native reindeer herders, and several were hospitalized. One proposed explanation for the outbreak was that warm temperatures melted permafrost, exposing the corpses of reindeer that had died of anthrax almost a 100 years earlier, and releasing infectious *Bacillus anthracis* spores into nearby waterways. However, another valid explanation is the large increase in reindeer herd sizes, discontinuation of routine anthrax vaccination of reindeer (110), loss of Indigenous Knowledge among herders, and lack of veterinary experience to recognize signs of the disease in reindeer (111). Certain zoonotic disease agents survive particularly well in cold northern climates, including spore-forming bacteria, *Mycobacterium* species, protozoan cysts/oocysts, some helminth eggs, prions, non-enveloped viruses, and pox viruses (281). Some fungi can survive in permafrost for extended periods, and nematodes can be viable after long-term cryobiosis in Arctic permafrost (310). Most viruses are rapidly inactivated outside host cells: while RNA from the 1918 influenza strain was detected from buried Arctic peoples almost 100 years after their death (18), the material was non-infectious. Giant viruses appear to be more resilient: a giant virus trapped for around 30,000 years was recently isolated from Siberian permafrost (311), and two plant viruses recovered from 700-yr-old caribou feces still demonstrated infectivity (312). In addition to revealing dormant pathogens, climate warming also leads to release of persistent environmental pollutants, heavy metals, carbon and other natural elements from soils and rocks (313). These have cumulative effects on the immune systems and health of wildlife and humans, increasing susceptibility to diseases, particularly when combined with additional climate-related stressors such as reduced or altered habitat and food availability for wildlife, and, in turn, for humans (186).

In terms of pathogens of epidemic or pandemic potential, climate-driven changes that influence wild Arctic water bird habitat use, distribution, and migration could be a factor in the global distribution of avian viral agents, and possibly the emergence of a new pandemic influenza strain (186). Several recent studies provide evidence that migratory birds serve as effective long-distance vectors of wildlife and zoonotic pathogens to the Arctic (282), with boreal regions providing stop-over sites for many migratory species (314–316).

Most vector-borne disease agents have limited pandemic potential, as their spread tends to be restrained by geographically and climatologically restricted vector habitats (317). However, with warming environmental temperatures, and changing precipitation levels, vector-borne pathogens will become of increasing concern for epidemics in Arctic and boreal biomes, as vector ranges expand. Such viruses include those spread through mosquito bites like West Nile virus and Sindbis virus, which are maintained in wild birds; California serogroup viruses, such as Jamestown Canyon virus (antibodies to which have been found in bison (*Bison bison*), Dall's sheep (*Ovis dalli*), snowshoe hare (*Lepus americanus*), and Arctic fox (*Vulpes lagopus*) in Alaska); and Snowshoe hare virus which is maintained in mammalian hosts including rodents, deer, and hares (186, 276). Tick borne-encephalitis virus can also infect multiple hosts including ungulates, birds, rodents, and carnivores, and is spread from animals to humans through tick bites. In the Russian Arctic, climate associated increases in mosquito and tick populations, and rodent-borne infections transmitted by arthropods are already having an impact on population health (281). Insect repellent and avoidance of tick bites will likely become increasingly important for people living in northern communities.

As existing agricultural regions are threatened by climate change, warming of high latitude regions, and increasing food demands may lead to northward expansion of global agriculture (318). Agriculture is associated with the emergence of more than 50% of zoonotic infectious diseases in humans (319), and future rates of zoonotic disease emergence and/or reemergence are predicted to be closely linked to the evolution of the agriculture–environment nexus (42, 202, 238, 320). As opportunities for agriculture, and other industries, increase with warming of Arctic and boreal biomes, there is potential for disruption and dramatic change of these ecosystems from an influx of people, domestic livestock, and pets, and the disease agents they harbor (226, 243, 321). Beyond prospective increases in infectious disease emergence, climate-driven agricultural expansion will have major impacts on biodiversity, on downstream water resources, and on carbon storage (322). Multiple links exist between human health and anthropogenic environmental degradation. Land-use modifications that lead to the loss of ecosystem integrity, and increased human-domestic animal-wildlife contact rates can increase susceptibility to emerging zoonoses directly, but also indirectly via impacts on the immune system, mental health issues, environmental contaminants, and endemic diseases, paired with reduced access to fundamental services such as timber, freshwater, wild foods, medicines, and decreased air quality (248). A systematic, interdisciplinary, and holistic approach to understand and address environmental health, zoonotic, and other disease concerns around agricultural and other developments are key to mitigating negative impacts (323–325).

### Current and Future Considerations for Monitoring, Surveillance, and Risk Reduction Approaches

Given the changing socio-ecological, -economic, and -political systems of Arctic and boreal biomes, future research would be beneficial to understand how the increasing anthropogenic impacts across northern regions are altering ecological processes, and thereby potentially converting microbial hazards in naturally occurring pathogen diversity into risks to human health (277, 326, 327).

Human health monitoring for zoonotic diseases in Arctic and boreal biomes is typically coordinated through relevant local health authorities and health surveys (e.g., Inuit Health Survey). Outbreaks can lead to new sampling programs, such as the sampling of walrus (*Odobenus rosmarus*) meat before sharing and distribution in the community (189). In general, there has been minimal research on the risks associated with traditional and country food preparation practices, despite awareness of zoonotic pathogens found in harvested wildlife and a clear need for risk reduction (118). Good hygiene during butchering and skinning, and thorough cooking of food can prevent transmission of many of the endemic zoonotic pathogens reported (118). Most health status measures and outcomes are consistently poorer for Indigenous Peoples in comparison to the rest of the North American population (63, 328) and investing in health infrastructure across Arctic and boreal biomes is critical to meet the broader needs of these populations. These investments would enhance emerging infectious disease surveillance, contribute toward a better understanding of the patterns of exposure and immunity, and reduce risks from both endemic and emerging zoonoses (79, 248, 329). Ensuring ownership of health initiatives by local communities in general, and specifically in the necessary prioritization process, is essential, with ample evidence demonstrating that failure to engage and build trust with local political and thought leaders, Elders, traditional health workers, and community groups in disease detection and control, will delay both diagnosis and response for emerging diseases (330). Identifying priority pathogens is a valuable starting point given present-day perceived financial constraints (281), though broader monitoring systems capturing syndromic trends may be more sensitive to detect emerging pathogens. Furthermore, every community has location-specific, individual risk profiles that will help determine the discrete approaches needed. To provide timely, accurate, and pertinent information on zoonotic disease risks across northern regions, the current capacity for northern communities to establish or continue disease monitoring and diagnosis needs to be expanded (186, 193, 331).

National, regional, state and territorial level veterinarians and programs monitor the health of wildlife across North American Arctic and boreal biomes. These programs range from highly standardized to less formal efforts, and vary in their objectives, from basic data collection, to in-depth species specific research. Recommendations regarding the reduction of disease risk from wildlife are detailed in a variety of local guidance publications (see Table 3). Furthermore, many groups publish regular or as-needed health updates through websites, co-management meetings, and scientific gatherings. The Canadian Wildlife Health Cooperative (332) forms a cross-Canada network of collaborators dedicated to wildlife health and a national surveillance program for wildlife diseases, but the approach to date has been essentially reactive to emerging issues. A new “Pan-Canadian Approach to Wildlife Health” was initiated in 2018 (333), and represents a deliberate transition to proactive health promotion; however, increased funding commitments, and jurisdictional collaboration and coordination, are necessary to enable its execution. In Alaska, the U.S. Geological Survey's National Wildlife Health Center (NWHC) (334) supports wildlife disease detection, control, and prevention, and conducts wildlife disease outbreak investigations as part of a national, general surveillance program. The Alaska Department of Fish and Game has a longstanding wildlife disease monitoring program and wildlife health monitoring also occurs at local levels: the North Slope Borough Department of Wildlife Management has multiple harvest monitoring programs for subsistence species [e.g., bowhead whale (*Balaena mysticetus*), beluga, ice seals (Phocidae), walrus, polar bears (*Ursus maritimus*)], and has a well-developed wildlife health research program for these mammals. This program, and an increasing number of other programs in northern regions, are community-based, and work with hunters and communities to answer questions about health and diseases in wildlife in a changing Arctic environment. Some examples of current monitoring institutions and efforts regarding health of wildlife and traditional foods are shown in Table 4.

**Table 3:** Examples of Existing Guidance on Safety for Hunters.

**Hunter guidance document and source**	**Reference and website for accessing document**
Safety manual for harvesters of fish and wildlife in nunavut	Canadian Wildlife Health Cooperative and Government of Nunavut. (2011). Safety Manual for Harvesters of Fish and Wildlife in Nunavut: An Illustrated Guide to Common Diseases and Parasites. https://www.gov.nu.ca/sites/default/files/files/Safety%20Manual%20for%20Harvesters%20of%20Fish%20&%20Wildlife%20in%20Nunavut.pdf (accessed March 10, 2021)
Disease precautions for hunters	American Veterinary Medical Association (2021). https://www.avma.org/resources/public-health/disease-precautions-hunters#protecting (webpage only) (accessed March 10, 2021)
Diseases you can get from wildlife	Government of British Columbia (2017). Diseases You Can Get From Wildlife: A Field-guide for Hunters, Trappers, Anglers and Biologists. https://www2.gov.bc.ca/assets/gov/environment/plants-animals-and-ecosystems/wildlife-wildlife-habitat/wildlife-health/wildlife-health-documents/diseases_you_can_get_from_wildlife_field_guide_2017.pdf (accessed March 10, 2021)
A field guide to common wildlife diseases and parasites in the northwest territories	Government of Northwest Territories (2017). A Field Guide to Common Wildlife Diseases and Parasites in the Northwest Territories'; 6th Edition, March 2017. https://www.enr.gov.nt.ca/sites/enr/files/field_guide_wildlife_diseases.pdf (accessed March 10, 2021)
Common wildlife parasites and diseases	Alaska Department of Fish and Game (2021). Common Wildlife Parasites and Diseases. Available online at: https://www.adfg.alaska.gov/static/home/library/pdfs/wildlife/brochures_newsletters/common_wildlife_parasites_diseases.pdf and https://www.adfg.alaska.gov/index.cfm?adfg=disease.main (webpage only) (accessed March 10, 2021)

**Table 4:** Examples of current health monitoring institutions and efforts for health of wildlife and traditional foods (not intended to be comprehensive).

**Institution**	**Website**
Alaska Native Tribal Health Consortium	https://anthc.org/what-we-do/traditional-foods-and-nutrition/
North Slope Borough Department of Wildlife Management	http://www.north-slope.org/departments/wildlife-management/studies-and-research-projects/health-assessment-of-subsistence-resources
Unusual Mortality Events (UMEs)	https://www.fisheries.noaa.gov/insight/understanding-marine-mammal-unusual-mortality-events
Community-Based Monitoring	https://www.inuitcircumpolar.com/wp-content/uploads/2019/01/cbm_report_final.pdf
LEO Network	https://www.leonetwork.org/en/#lat=60.71611&lng=-135.05375&zoom=7
Canadian Wildlife Health Cooperative	http://www.cwhc-rcsf.ca/
US Geological Survey (USGS) National Wildlife Health Center	https://www.usgs.gov/centers/nwhc
US National Oceanic and Atmospheric Administration (NOAA)	https://www.fisheries.noaa.gov/national/marine-life-distress/marine-mammal-health-and-stranding-response-program
USGS Alaska Science Center	https://www.usgs.gov/centers/asc/science-topics/wildlife-disease

Community-based monitoring, Indigenous Knowledge, participatory epidemiology, and citizen science have become increasingly relevant as ways of centering research on community needs and priorities, and obtaining invaluable information on health and the environment (190, 193, 197, 201, 331, 335–338). Rich knowledge, including on preventing certain food-borne illness, results from the long-standing relationships of Indigenous Peoples and local communities with their environment, including their harvesting of nutritionally and spiritually important native plants, fish, and wildlife (56, 57, 118, 339, 340). Indigenous knowledge; the prevention, monitoring, and surveillance of zoonotic agents; and education are considered the most important methods to reduce human health risks associated with the consumption of traditional and country foods (118). Many wildlife harvesters in Arctic and boreal biomes are keenly aware of concerns regarding wildlife health and food safety. In general, parasites, lesions (discoloration, tracts, fungal growths, etc.), or abnormal behavior of hunted animals are often observed by Indigenous and local communities. Indigenous Peoples were some of the first to draw attention to traditional and country food safety concerns related to environmental contaminants, because of changes they detected in quality of the animals and fish they hunted (118). Community-based monitoring programs contribute valuable information. For example, the Nunavik Trichinellosis Prevention Program provides rapid carcass-side testing for trichinella in country foods for Nunavik communities (341), giving community members control to obtain the information they need to make informed decisions about food preparation, consumption, and carcass disposal to prevent further transmission. Recent working groups assessing Unusual Mortality Events (UMEs) for marine mammals and other species around Alaska include Indigenous perspectives and observations (191, 342) and, across Canada, several Indigenous-led Guardians Programs monitor ecological health, maintain cultural sites, and protect sensitive areas and species, while also playing a vital role in creating conservation plans, and supporting Indigenous Protected and Conserved Areas (343). Table 3 lists some examples of the existing monitoring efforts for wildlife health, and country and traditional foods. Ongoing academic-government-community collaborative partnerships have resulted in several successful community-based wildlife health surveillance programs that aim to address community concerns about wildlife health and food safety while simultaneously addressing key research questions about wildlife ecology and dynamics (193, 197, 338).

Inuit, Métis, First Nations, and Alaskan Native societies across the Arctic and boreal biomes (and globally) maintain vast and holistic Indigenous Knowledge Systems, across generations, about the natural environment and how it is changing. Such awareness and presence of eyes-in-the-field is irreplaceable for the early detection of changes in wildlife populations, and the environment. Combining Indigenous Knowledge with scientific understanding improves wildlife surveillance, fosters reconciliation, and advances Indigenous Peoples' self-determination in research, while creating mutual health and conservation benefits (331, 344–348). However, a history of colonialism, relocations, residential schools, and loss of Indigenous languages has led to generational changes in diet, life-style and relationships with the environment for many Indigenous communities. Loss of transgenerational “hands-on” and oral transmission of knowledge, especially regarding harvesting, butchering, food safety and zoonoses, may increase zoonotic risk, increase meat wastage, and limit the ability to engage with “two-eyed-seeing” within participatory epidemiology (331). In addition to fostering reconciliation with Indigenous Peoples, programs that assist Elders to pass on Indigenous Knowledge to Indigenous youth, and brings communities together to prepare and share country and traditional foods, contributes to future monitoring of wildlife, human and environmental health, and supports a conservation economy focused on the land and wildlife. The participation and leadership of men and women involved in the harvest and preparation of wild foods, and youth in wildlife and community health monitoring and research, builds trust between researchers and local communities, particularly when focused on community-raised concerns and priorities (197, 331). Continued investment in existing community-based wildlife and human health monitoring efforts, paired with consistent and well-conveyed methodologies, would be a valuable approach to tracking wild harvest use and trade, zoonotic and other emerging pathogens, host species, and environmental impacts from the changing climate across northern communities.

Early recognition and intervention during an emerging infectious zoonotic disease event is essential to limit spread (349). Wild animals, and domestic dogs, can serve as sentinels for zoonotic diseases and other health concerns such as contaminants and it appears essential that existing veterinary and wildlife surveillance systems for zoonotic pathogens are closely integrated with public health surveillance, to better control such pathogens before they affect human health. Surveillance would benefit from the exploration and cooperative development with local and Indigenous leaders of practical ways to integrate and share surveillance-generated information for humans, domestic animals and wildlife. This would ensure that the surveillance systems are risk-based, spatially and temporally targeting species and geographic locations with the highest risk for spillover, and guaranteeing that the information gathered can be used by the local communities to deploy interventions in a timely manner. Expansion of regional, transdisciplinary One Health networks that involve community members and diverse stakeholders and collate real-time multifactorial data and explicit observations, (e.g., the Echo Network for health and conservation, the Circumpolar Climate Change and Infectious Diseases Workgroup, or the LEO network^1^) (350) and integration with public health networks such as the International Circumpolar Surveillance (ICS) project (192), can facilitate early-warning systems, rapid response, and mitigation for health threats to animals, humans, and the environment, while also advancing our understanding of endemic, and emerging zoonotic health risks for these communities.

It is important that the desire to increase awareness of the potential risk of zoonotic infections be balanced with the unintended consequences on other determinants of health. Past experience with poorly designed communication strategies and materials with respect to country and traditional foods, such as contaminants in wild-caught foods, provides stark lessons on the need to avoid provoking unnecessary fear when addressing food safety risk in Indigenous and local communities (71, 72). For example, scientific communication to Nunavut Inuit communities in the late 1980s that women's breast milk had high levels of polychlorinated biphenyls (PCBs), failed to consider broader understanding of contamination issues in the region, and failed to include the Inuit directly on messaging (72). The resulting alarm and confusion across Inuit and other northern communities led many women to stop breastfeeding, and stopped consumption of country and traditional foods, leading to other more insidious health issues, poor nutrition, and food insecurity (72). Similar issues were associated with the research and communication of contaminants in wildlife in Alaska. Participation in subsistence activities, including the practice of hunting by Indigenous Peoples, is considered a protective mental health factor for Indigenous circumpolar youth (351), and there are different and pertinent health concerns associated with the consumption of store-bought, processed, imported market foods increasingly eaten by younger generations. Such foods are often low in nutritional value, high in sugar, and sometimes harbor contaminants including microplastics and pesticides (56, 352–354). In Alaska, the introduction of modern materials such as plastic bags, plastic pails and glass jars in the preparation of rendered and aged foods (*muktuk* and *igunaq)* actually increased outbreaks of botulism (118). Indigenous and non-Indigenous health professionals promoted the return to traditional methods thereby reducing fatality rates by leveraging knowledge from community members, Elders, and survivors of botulism. Communications and management approaches around zoonotic disease risk from traditional and country foods are better co-developed with Indigenous and other hunting/trapping communities, with mindfulness that hunters and their families are keenly aware of changes in the behavior of wildlife or condition of wildlife products; these foods are usually the most cost-effective and healthy sources of nutrition in northern and remote communities; and country and traditional foods have important cultural, spiritual, and social values to northern communities (355, 356).

Combining diverse approaches and ways of knowing, including Indigenous Knowledge, provides a more complete understanding of the socio-ecological system than applying Western science alone. Working together can help bridge gaps in scientific monitoring, and bring the best available knowledge to more effectively monitor, and respond to, the impacts of disease and climate change on the health of Arctic and boreal inhabitants (338).

## Conclusion

An essential connection exists between subsistence activities, and well-being and resilience within Indigenous Peoples and local communities across the Arctic and boreal biomes in North America. The current threat of pandemic zoonotic disease emergence from hunting, consumption, and use of wildlife in the North American Arctic and boreal biomes is low, and policies restricting these traditional and subsistence activities in the name of pandemic prevention would be greatly misplaced. Health threats from endemic zoonotic diseases in northern biomes remain, with plausible hypotheses that environmental alterations from development, and climate change could alter epidemic and pandemic risks. Whilst commercialization of trade in wild food and products, and agricultural expansion have been identified as factors important in developing resilience in the North (351, 356, 357), they will bring a different set of challenges around zoonotic disease emergence, and disruption of ecosystems (238, 323). Co-development of any future policies and interventions with Indigenous Peoples and local communities may more effectively address zoonotic disease emergence in these rapidly changing northern regions. Combining Indigenous Knowledge Systems with science provides a more holistic understanding of zoonotic risks in Arctic and boreal ecosystems, informs opportunities for mitigation and monitoring, and improves disease risk communications, while avoiding potentially negative repercussions (331, 356, 358, 359). Monitoring, research, and response efforts would all benefit from employing more inclusive One Health approaches that draw on all knowledge systems and types of expertise, and proactively incorporate the complexity and interrelatedness of the environmental, biological, economic, political, cultural, and social dimensions of zoonotic disease emergence in northern biomes.

## Author Contributions

LK, CW, MR and JR contributed to conception and design of the study. LK organized the database, performed the review and analysis and wrote the first draft of the manuscript. MR and C-LC also wrote sections of the manuscript. All authors contributed to manuscript revision and additions, further development of the research concept and read, and approved the submitted version.

## Conflict of Interest

DJ was employed by the company Nyati Health Consulting, British Columbia, Canada. DL was employed by Nunavut Tunngavik Inc., Ottawa, Canada. The remaining authors declare that the research was conducted in the absence of any commercial or financial relationships that could be construed as a potential conflict of interest. The reviewer AP declared a past co-authorship with one of the authors SO to the handling Editor.

## Footnotes

^1^The LEO Network collates and shares information on unusual events, climate and other drivers of environmental change. The online platform is multi-lingual, including Indigenous languages, and was designed to be accessible from remote locations, in order to gather input from local community members, using holistic observational data, and welcoming diverse knowledge systems. The network facilitates connectivity between communities, environmental and public health experts, and allows researchers and agencies to use data as a surveillance system for local updates on emerging issues and vulnerabilities, and to provide event specific consultations.
