# Supplementary material for: Implications of Zoonoses From Hunting and Use of Wildlife in North American Arctic and Boreal Biomes: Pandemic Potential, Monitoring, and Mitigation
Source: Front Public Health. 2021 May 5;9:627654. doi: 10.3389/fpubh.2021.627654 (PMC8131663; doi:10.3389/fpubh.2021.627654)
Supplement: Supplementary file 1 [file Data_Sheet_1.PDF]

Appendix 1: Brief Summary of Zoonotic Diseases with Potential for Transmission to Humans Through the Hunting, Consumption, and Use of Wildlife in Arctic and Boreal Biomes

Zoonoses can be eukaryotic parasites, viruses, bacteria, prions, and fungi. A eukaryotic parasite is an organism that lives on or in a host organism and gets its food from or at the expense of its host. The three main classes of disease-causing parasites in humans are protozoa, helminths, and arthropods. A virus is an obligate intracellular parasite, too small to be seen by light microscopy, and able to multiply only within the living cells of a host. Bacteria are microscopic, single-celled organisms that can exist both as free-living organisms or reliant on a host. Fungi are spore-forming eukaryotic organisms that feed on organic matter. This Appendix provides a brief summary of zoonotic diseases reported in Arctic and boreal regions that can be transmitted to humans via the hunting, consumption or use of wildlife.

Parasitic Diseases

Anisakidosis

Anisakidosis in humans is caused by nematodes (roundworms) from the genus *Anisakis*, that burrow into the stomach wall and cause ulcerative lesions. Bearded seals (*Erignathus barbatus*), ringed seals (*Pusa hispida*), and beluga whales (*Delphinapterus leucas*) are likely definitive hosts for these parasites in northern hunting grounds, with crustacean zooplankton, fish, and squid acting as intermediate hosts (73). Consumption of raw fish poses the main risk of infection to humans (74). Uncooked whale or seal intestine could potentially pose concern but has not been documented. Individuals can also develop long-term sensitivities to larval Anisakis, thereby depriving them of fish in their diets. With respect to disease risk associated with Inuit country foods, among the species of fish examined, Atlantic tomcod (*Microgadus tomcod*), polar cod (*Boreogadus saida*), and sculpins (*Cottoidea*) represented the greatest risk of foodborne disease from *Anisakis simplex* and *Pseudoterranova decipiens* (73).

Fish-borne Fluke Infection

Fish-borne trematodes (fluke) infect the liver and intestines of humans and mammals worldwide, but only one human infection has yet been reported in the Arctic: a person infected with the heterophyid fluke *Cryptocotyle lingua*, in Greenland (75). This case was associated with the ingestion of raw or improperly cooked fresh or brackish water fish containing infectious metacercariae cysts. Various fish-eating birds and mammals in Northern Europe have been identified as reservoir hosts, including foxes (*Vulpes* spp.), gulls (*Laridae*), terns (*Sternidae*), and herons (*Ardeidae*) (76).

Cystic and Alveolar Echinococcosis

Echinococcus canadensis

In northern Canada, two genotypes of the tapeworm *Echinococcus granulosus* (G8 and G10), unified under the name *Echinococcus canadensis*, circulate in largely sylvatic cycles involving cervids and wild canids, and only the G8 genotype has been reported in Alaska (22,79). *E.*

canadensis causes cystic hydatid disease in humans and, in northern regions, caribou/reindeer (*Rangifer tarandus*), moose (*Alces alces*), muskoxen (*Ovibos moschatus*), and deer (*Cervidae*) serve as intermediate hosts for the parasite (78-80). Intermediate hosts carry the larval forms in their liver and lungs, and definitive canid hosts, e.g. dogs (*Canis lupus familiaris*), wolves (*Canis lupus*), foxes (*Vulpes* spp.), coyotes (*Canis latrans*), are infected through the consumption of infected organs of an intermediate host, and carry the adult parasite in their intestine (77,79,81–83). Humans become infected with *Echinococcus* spp. unknowingly and incidentally via the accidental ingestion of eggs shed in feces of a canid host, e.g. from eggs stuck to fur during fox trapping and skinning and, particularly if skinning is performed in the home, from desiccated eggs that can become aerosolized (82); or from an infected water or food source contaminated with eggs. The infection caused by *E. canadensis* in humans develops slowly and results in cyst formations in the liver and lungs.

Patterns of cystic echinococcus endemicity have been associated with domestic dog management in the Arctic, including cycles involving herding-dogs and domesticated reindeer in arctic Eurasia; sled-dogs of Indigenous hunters consuming the lungs of wild reindeer in Alaska; and occurrence of cystic echinococcosis among nomadic Arctic peoples increased when their communities became sedentary, and their village environment was contaminated by dog feces, with prevalence decreasing once sled-dogs were replaced by machinery (78).

Alveolar echinococcosis

In contrast to the relatively mild human disease caused by *E. canadensis*, alveolar hydatid disease caused by *Echinococcus multilocularis* can cause parasitic cysts in the liver, lungs, brain, and other organs and is associated with a much higher mortality rate. *Echinococcus multilocularis* maintains a life cycle that includes dogs (*Canis lupus familiaris*), wolves (*Canis lupus*), foxes (*Vulpes* spp.), coyotes (*Canis latrans*) or felids as the definitive host, with an intermediate host such as a vole (*Microtus* spp.) or a deer mouse (*Peromyscus maniculatus*) (79,84). Humans become infected via the accidental ingestion of eggs shed in feces of a canid host, as for *E. canadensis* during handling of a canid or canid fur, or from an infected water source or plant material contaminated with eggs. Early diagnosis is critical for more effective treatment of the disease in humans and rapid, simple serological tests are now available that can be used to screen at risk communities and identify asymptomatically infected individuals (85).

Other tapeworm infections

Diphyllobothriosis is a reemerging disease caused by *Diphyllobothrium* tapeworms. There are at least three *Diphyllobothrium* species of Arctic tapeworm reported in salmon (*Salmonidae*) in Alaska that can cause diarrhea or asymptomatic infection in humans: *Diphyllobothrium latum, Diphyllobothrium dendriticum,* and *Diphyllobothrium nihonkaiense* (86). Fish-eating mammals and birds are the hosts of the adult tapeworm, with the flesh of fish being the source of the infective life stage of the parasite. Humans become infected with *D. latum* through consumption of undercooked meat from wild fish containing the larval form. Infections by *D. dendriticum* are mainly acquired by the consumption of fish livers or stomach tissue in which the parasite is encysted and difficult to detect. *Diphyllobothrium dendriticum* could also migrate in the flesh if fish have remained long enough in nets. Infective stages can be destroyed by cooking fish and

meat to at least 55 °C for 10-30 minutes or freezing at -18 °C for 24 hours (73). Drying eventually kills the parasites, but cold-smoking and other preservation methods likely do not.

Toxocariasis

Toxocara canis and *Toxocara cati* are zoonotic nematodes with *T. canis* primarily transmitting amongst canids (e.g., dogs, wolves, foxes, and coyotes) with small mammal paratenic hosts, and *T. cati* transmitting mainly between felids e.g. lynx (*Lynx canadensis*) and wild rodents. These nematodes are responsible for ocular and visceral larval migrans in people, and have been reported in boreal biomes of northern Canada and Alaska (79,88,89). Their presence is not well documented in the Arctic across North America; however, a 2008 study (87) found prevalence of *T. canis* in domestic dogs to have increased in Alaska over the past few decades, suggesting that this parasite may be becoming more successful at Arctic latitudes. Other ascarid nematodes reported in carnivores in the North include *Toxascaris leonina* (83) and *Baylisascaris* spp.; however, their zoonotic potential is considered low (90). Accidental ingestion of eggs (shed by definitive canid and felid hosts) in a contaminated environment is the main route of infection for people, though acquisition could also occur through consumption of uncooked meat of small mammal paratenic hosts (82). Human infection could potentially occur via infective oocysts during the skinning process, as has been reported for *Echinococcus* species (82).

Toxoplasmosis

Toxoplasmosis is caused by the protozoan parasite *Toxoplasma gondii.* This disease can be contracted through the consumption of raw or undercooked meat that contains parasite tissue cysts, or food, water, or soil contaminated with cat feces (the definitive host) containing infective oocysts (79,178). Antibodies to *T. gondii* have been reported in terrestrial and marine mammals and birds, and people throughout northern Canada and Alaska, and have also been reported in wildlife (muskoxen, and polar bears (*Ursus maritimus*)) in eastern Greenland (178,187). Filter-feeding invertebrates and fish may filter and concentrate infective oocysts from the marine environment, and remain infective for at least 8 h (188). The disease in humans is often mild or asymptomatic, though there are more recent postulations of potential association with mental health issues such as mild depression and schizophrenia (98,99). In immune-compromised individuals, more severe complications such as encephalitis or acute chorioretinitis may occur. However, the main concern is for pregnant women as infection can cause fetal morbidity and mortality (84, 91).

People in northern communities who practice subsistence hunting often have an increased infection risk due to traditional food preparation techniques, such as consumption of raw meat and frequent handling of wild game (100). Many northern animals and people demonstrate relatively high exposures to *T. gondii*: a survey of Nunavik Inuit showed a seroprevalence of 60%, almost three times the North American average prevalence. The low densities of domestic and wild felid hosts in this region suggest alternative transmission mechanisms: a multivariate analyses identified consumption of seal meat and wild birds as risk factors for seropositivity (92). It is likely that the source of infection is related to the consumption of raw, dried or undercooked meat e.g. of caribou (*Rangifer tarandus*), walrus (*Odobenus rosmarus*), seals, waterfowl, and other birds (79). Hunters and trappers would benefit from taking special precautions when hunting, trapping, and handling lynx, as these felids serve as the only known Alaskan wildlife source of *Toxoplasma* sp. oocysts (84). For boreal communities, *T. gondii* infection is prevalent in black bears (*Ursus americanus*)

and white-tailed deer (*Odocoileus virginianus*). Exposure levels for the parasite can be high in these species, and human infection as a result of the consumption of undercooked bear meat or venison has been reported (101). Wild geese in Svalbard and the Canadian Arctic have been identified as likely vectors for transmission of *T. gondii* to Arctic foxes (*Vulpes lagopus*), polar bears, marine and other mammals in the Arctic food web (95,100). *Toxoplasma gondii* may also be a direct concern for wildlife population health, as the parasite causes abortion and congenital disease in many animal species, including captive reindeer (93), has led to die-offs of arctic foxes elsewhere in the Arctic (119), and may be influencing population declines of species important to food and economic resilience of Indigenous communities, such as caribou (79).

Trichinellosis

Trichinellosis, also called trichinosis, is caused by roundworms in the genus *Trichinella*, that live in the muscle tissue of infected animals. *Trichinella* species in more temperate regions (e.g., *T. spiralis* and *T. pseudospiralis*) can be killed by freezing, whereas the species in northern regions (*T. nativa* and *Trichinella* genotype T6) are freeze tolerant. Illness in humans is variable ranging from asymptomatic infection to a rarely fatal disease, depending on the number of larvae ingested (84). Walrus, seal, and bears (*Ursidae*) eaten by many Arctic and some northern boreal communities are known hosts of *Trichinella nativa* and T-6 (30,84,79,82,102-104). Wolverines (*Gulo gulo*) in northern Canada were recently found to be infected with *Trichinella pseudospiralis*, which causes clinical manifestations in humans similar to those caused by *T. nativa* except with a more prolonged myopathy (105). The infective stages of *Trichinella* parasites are microscopic in size, and thus can easily be unintentionally ingested in raw or undercooked meat. Walrus is often eaten raw, undercooked, or processed into a fermented product, with none of these preparation methods killing the parasite. Fox, wolf, and wolverine are known hosts for *T. nativa* (previously thought to be *T. spiralis*). Polar bear has a *Trichinella* seroprevalence in some regions of 60% (82,106). These species are often hunted for fur and, when eaten, carnivores are typically cooked, which should kill the infective parasite.

Giardiasis and Cryptosporidiosis

Giardiasis, caused by *Giardia intestinalis*, *G. lamblia*, or *G. duodenalis* and Cryptosporidiosis, caused by *Cryptosporidium parvum* or *C. hominis* are protozoal diseases that usually cause diarrheal disease in humans. Humans contract these pathogens through water contaminated with feces containing infective oocysts shed by animals, typically beaver (*Castor canadensis*) and muskrats (*Ondatra zibethicus*), with transmission not directly related to subsistence harvesting, though *Giardia* sp. have been isolated from seals slaughtered by Inuit hunters (107).

Bacterial Diseases

Anthrax

Anthrax is caused by infection with the bacterium *Bacillus anthracis*. The disease most typically affects wild and domestic ungulates, and is usually rapidly fatal, with mammalian predators and scavengers often also affected. The disease is zoonotic and in people ranges from a localized skin infection to fatal disease following ingestion or inhalation, or contamination of wounds by

bacterial spores. Anthrax has so far affected relatively few wild animals in the Arctic and boreal regions of North America, but has been recognized as an important cause of mortality in bison in northern Alberta and the southern Northwest Territories (108). Wild ungulates (deer, elk and bison) have also died in association with outbreaks of anthrax in domestic cattle (*Bos taurus*) in Saskatchewan (109). In Canada, outbreaks have been linked to intense precipitation, droughts, and other climatic factors, in particular wet springs followed by hot, dry summers (108). Emergence of anthrax in the Russian Arctic recently caused disease outbreaks in reindeer and humans, (110,111) and is discussed as a future concern in the main narrative.

Brucellosis

Brucellosis is caused by *Brucella* spp. and the bacteria have been isolated from many species of wild mammal, including caribou, muskox, bison (*Bison bison*), wolf, fox, wild rodents, hares (*Lepus* sp.), deer, and other ungulates (81,112). Free-ranging bison are the known wild reservoir for bovine brucellosis (*B. abortus*) in Canada (113). *Brucella suis* biovar 4 is enzootic in caribou/reindeer and care should be taken when handling caribou (and other ungulate) carcasses, fetuses, and newborn calves. Risk of acquiring *Brucella* spp. infection can be reduced in endemic areas by avoidance of feeding raw caribou or other ungulate meat or entrails to dogs, and of the consumption of raw (including frozen or dried) caribou meat and marrow. Brucellosis in humans is a systemic bacterial disease that can have an acute or gradual onset with intermittent fever of variable duration with headache, weakness, sweating, chills, joint pain, and weight loss (84). Marine mammals can also be affected or carry *B. pinnipedialis* and *B. ceti.* Marine *Brucella* spp. have rarely caused neurobrucellosis in humans, following the consumption of uncooked fish (114).

Botulism

Whilst not infectious, thus not a zoonosis in the strictest definition, botulism is connected to the consumption of fermented meat products, and Indigenous Peoples are the highest-risk group for botulism, so it warrants mention (115,118). Toxins produced by the bacterium, *Clostridium botulinum*, cause multiple clinical symptoms in humans such as blurred vision, nausea, vomiting, paralysis of the motor nerves, and respiratory paralysis in fatal cases (119). Botulism is related to handling and storage of food, as well as certain food preparation habits: 60% of registered Canadian botulism outbreaks from 1971 to 1984 were caused by meats from marine mammals, mostly seals, that were consumed raw or parboiled, or had undergone a fermentation process (i.e., *igunaq*, *utjaq*, and *muktuk*) at higher temperatures (114–116). All outbreaks occurred among Indigenous communities. An outbreak of human botulism in Greenland in 1990 was associated with consumption of raw seal meat and intestines. Use of plastic containers to prepare or store fermented food favors the growth of *C. botulinum* as do fermentation processes that use higher temperatures or longer incubation times (117). This toxin is inactivated by thorough cooking of the meat.

Erysipeloid

The zoonotic bacterial pathogen *Erysipelothrix rhusiopathiae* has been associated with recent mortalities in Arctic and boreal ungulate populations (including muskoxen, deer, and caribou) and is considered an emerging pathogen in these regions (120,121,122,127,128). Generally considered an opportunistic pathogen of domestic swine and poultry, *E. rhusiopathiae* can infect multiple

hosts and has been found in terrestrial and aquatic mammals, birds, fishes, and arthropods (124). Transmission of *E. rhusiopathiae* to humans occurs primarily through exposure to animals or animal products infected with the organism, via non-intact skin or ingestion, with environmental sources of infection also reported. *Erysipelothrix rhusiopathiae* infection in humans may also be acquired through contact with infected fish via open skin wounds (126).

In people, it most commonly causes erysipeloid, a localized cutaneous infection; however, more severe cases with diffuse cutaneous or systemic disease leading to septicemia and sometimes endocarditis can occur (128). Other infrequent manifestations include pneumonia, abscesses, meningitis, and septic arthritis, which has been reported in several native people, including an Inuit woman in the Canadian Arctic who regularly butchered and consumed raw wild meat (including caribou and muskoxen) and had no contact with domestic sources of infection (124). Until recently, reports of mortalities in wild ungulates were rare. Environmental and climatic changes may be increasing the severity and prevalence of *E. rhusiopathiae* in certain wild animal host populations, including multiple and large-scale die-offs of muskoxen in Nunavut, the Northwest Territories, and Alaska, and moose and caribou in British Columbia. In addition to the health risk of infection for people who interact with these animals or their environments, there is potential for reduced food security for hunting communities through the direct loss of animals from *E. rhusiopathiae* (56,120,123). Guidelines for hunters across northern Canada recommend that animals found dead not be touched or eaten, advise against cutting into animal parts that look abnormal, and whenever in doubt, to cook meat or fish well (125).

Leptospirosis

Leptospirosis is caused by bacteria in the genus *Leptospira*. The disease can present with a wide range of symptoms in humans, from none at all to kidney damage, meningitis, liver failure, respiratory distress, and even death if left untreated. Humans become infected through direct contact with contaminated urine or animals. Leptospirosis has been detected in beavers, coyotes, deer, foxes, opossums (*Virginia opossum*), otters (*Lontra canadensis*), raccoons (*Procyon lotor*), and skunks (*Mephitis mephitis)* in Canada (129) and has been found among northern fur seals (*Callorhinus ursinus*) in the Bering Sea (130). Sylvatic exposures have been reported among hunters, trappers, and Indigenous Peoples, with the most northern exposure identified in the Nunavik region of Québec (131).

Lyme Disease

Borrelia burgdorferi-infected ticks (the agents of Lyme disease in humans) have been found on sea-birds in the North Atlantic (132) and on songbirds in northern Canada (133). Although implications for human health in Arctic and boreal regions are unclear at this time, infection can cause fever, rash, facial paralysis, and arthritis, and vector-borne zoonotic diseases such as Lyme disease will likely become more important as the regional climate changes. An upward trend in tick-borne encephalitis in the northern European Arctic has been noted, and cyclical cases are also reported from the Russian Arctic (177). Care to avoid exposure to bites from ticks on hunted wildlife (birds and mammals) should be taken.

Pasteurellosis

Pasteurellaceae bacteria are a part of the normal oral flora in pinnipeds (135), and can cause septicaemia, mortality, and pneumonia in pinnipeds and cetaceans. *Pasteurella multocida* is a commensal in the upper respiratory system of many animal species, and also causes avian cholera. Transmission to humans occurs primarily through animal bites or contact with nasal secretions. Infection causes skin and soft tissue infections with rapidly spreading edema, erythema, and tenderness at site of bite and lymphadenitis. Guano is another potential source of *P. multocida*: it has been isolated from the cloaca of web-footed birds in Denmark, and pasteurellosis in seals in the United States has been associated with waterfowl migration (136,137). A novel member of the family Pasteurellaceae (*Bisgaardia hudsonensis*) was cultured from a person with an infected seal bite wound (134). Although infections in humans are primarily the result of bites from captive pinnipeds, *Pasteurellaceae* potentially pose a risk to those handling wild marine mammals.

Q Fever

The causative agent of Q fever is *Coxiella burnetii*, which has been detected in northern fur seals, with genetic sequencing indicating that seabirds play an important role in its regional distribution (139,140). A study by Miernyk et al. (138) found that Alaska Natives were almost three times more likely than non-Native Alaskans to be seropositive to *C. burnetii.* Humans normally acquire Q fever through inhalation of dust contaminated by infected animal feces, urine, milk, or birth products; or ingestion of infected animal products, e.g. milk or cheese. Mild or severe disease are possible, with symptoms that can include fever, fatigue, headache, muscle aches, nausea, vomiting, diarrhea, chest or stomach pain, weight loss, and cough. Pneumonia or hepatitis is seen in severe cases, and infection during pregnancy can cause miscarriage, stillbirth, preterm delivery, and low infant birth weight.

Seal Finger (Marine mycoplasmosis)

First described in 1907, Seal Finger is caused by infection with a *Mycoplasma* spp. bacteria and is most often seen in subsistence seal hunters, and professionals handling live seals. Infection normally occurs from seal bites and causes a swollen, painful, and suppurative lesion on the finger; however, one systemic case with fever and lymphangitis has been reported (114,141). Another bacteria *Bisgaardia hudsonensis*, a new member of the family *Pasteurellaceae*, was isolated from a person's infected seal bite in 2011 (134) and there may be additional emerging bacterial etiologies of this condition in humans.

Tuberculosis and Mycobacteriosis

Tuberculosis is a chronic infectious disease caused by bacteria from the *Mycobacterium tuberculosis* Complex (MTBC). *Mycobacterium pinnipedii*, which is known to cause disease in humans, has been isolated from different otariid (eared seal) species and isolated cases of dolphins (*Delphinidae*) in the wild, essentially in the Southern Hemisphere, as well from captive settings (114). An isolated case of wound infection by *M. bovis* has also been reported in a grey seal (*Halichoerus grypus*) (143). Since transmission to humans from captive seals has occurred, there is potential for wild seals and cetaceans to pose a risk of infection to hunters and people preparing these species for food. However, these risks in northern communities are most likely very low, because the prevalence of *Mycobacterium* from the MTBC seems to be almost nonexistent in marine mammals from this region. On the other hand, *M. bovis* infections have been reported in

six free-ranging species of terrestrial mammals in Canada (144), all of which are hunted by First Nations for either food or fur: bison, elk (*Cervus elaphus canadensis*), moose, white-tailed deer, mule deer (*Odocoileus hemionus*), and wolf. Humans can be infected by multiple routes including inhalation, ingestion (of raw/undercooked meat or unpasteurized milk products), and direct contact with mucous membranes or breaks in the skin. First Nations have acquired both pulmonary and cutaneous mycobacterial disease through dressing and butchering infected ungulates, and wearing protective equipment during these activities is recommended (125, 144).

Tularemia

Tularemia is a disease caused by the bacterium *Francisella tularensis*, which can be harbored in several species of northern wildlife including muskrat (*Ondatra zibethicus*), beaver, hares (*Leporidae*), voles (*Microtus* spp.), squirrels, wolves, and bears (142,145,146). Sporadic outbreaks are reported in trappers. People become infected through consumption of insufficiently cooked meat or contaminated water and dust. Infection can also be acquired through bites from infected vectors such as mosquitoes and ticks (145,146,147), and potentially via infected hunting dogs (148). Cooking meat, washing hands, using insect repellent, and removing observed ticks, which may become more prevalent as temperatures in the region rise, can help reduce risk of infection. Clinical presentations in people include skin lesions or ulcerations, lymphadenomegaly, vomition, abdominal pain, diarrhea, conjunctivitis, pneumonia, septicemia, and hepatospenomegaly (84).

Yersiniosis

Yersiniosis causes fever, abdominal pain, and diarrhea in humans. At least two species of Yersinia bacteria, - *Y. pseudotuberculosis and Y. enterocolitica* - can be zoonoses in northern Canada and the bacteria survive well in refrigerated conditions. Transmission to humans occurs through ingestion of undercooked meat and water contaminated by feces of infected animals. Careful hygiene is key to controlling infection and spread of the bacteria. Symptoms include gastrointestinal pain, diarrhea, and fever. The principal reservoir for *Y. entercolitica* appears to be domesticated swine, but the bacterium can survive in soil and water and is found in wildlife such as beavers and birds. *Y. pseudotuberculosis* is widespread in rodent and lagomorph hosts from which humans can become infected (142). Yersiniosis (*Y. pseudotuberculosis*) was found to be the proximal cause of death via enterocolitis in a die-off of muskoxen in Arctic Canada (149,151) and has been detected in Arctic foxes in Norway (348). Only two cases of *Yersinia pestis* have been reported from wildlife in Canada, in bushy-tailed woodrats in British Columbia in 1988 and in a prairie dog in 2014 (149,150). Based on surveys, *Y. pestis* may occur enzootically in southern Alberta, Saskatchewan, and British Columbia and if changes in climate lead to northern expansion of rodent reservoirs, the disease could affect Arctic and boreal communities (150).

Viral Diseases

Avian Influenza

Wild birds are the natural reservoir for low pathogenic avian influenza A viruses. Highly Pathogenic Avian Influenza (HPAI) viruses that emerged in poultry have been detected in wild birds in Asia since 2005–2006. Major migratory bird flyways exist between Asia and North

America and such flyways raise the possibility for the introduction of potentially pathogenic influenza viruses among wild birds and potentially to humans that hunt them and/or prepare them for eating (e.g., plucking, cleaning, butchering) (152). In 2016, a HPAI H5N2 virus was identified in a wild mallard duck (*Anas platyrhynchos*) from a state wildlife refuge near Fairbanks, Alaska (153). Although HPAIs have not yet been detected in people in the North American Arctic or boreal regions, hunting or consumption of obviously sick birds should be avoided and, when preparing waterfowl, frequent hand-washing and wearing face coverings to avoid any potential virus inhalation would be advisable. Influenza infection can cause mild or severe illness, and sometimes death with symptoms including fever, chills, cough, sore throat, nasal congestion, muscle or body aches, headaches, fatigue, vomiting, and diarrhea (the latter more common in children). Influenza A viruses have also been isolated from marine mammals, including whales and seals. Characterizations of these isolates found them to be avian-like, supporting the theory of interspecies transmission of influenza A virus from avian reservoir hosts to marine mammals (154). Transmission of influenza virus from marine mammals to humans has been documented. The virus caused conjunctivitis in people who acquired infection from handling sick and dead seals during the Influenza A epidemic in the United States in 1979 (155). Whilst not yet reported in the Arctic, avian influenza viruses could potentially infect and cause disease in humans through marine mammals (154).

Avian influenza viruses do have the potential to cause epidemics and evolve into human viruses with pandemic potential, as well as cause morbidity and mortality in people. Butchering or consumption of seals or whales and birds found apparently sick or dead should be avoided.

Calicivirus infections

Infection with caliciviruses has been documented in a wide range of Arctic marine mammals, including seals (e.g., fur (*Callorhinus ursinus*) and elephant (*Mirounga angustirostris*)), walrus, and whales, (including bowhead (*Balaena mysticetus*) and grey (*Eschrichtius robustus*)) (114). San Miguel sea lion calicivirus was isolated from fluid-filled blisters on the extremities of a marine mammal researcher exhibiting flu-like symptoms, indicating that marine mammal caliciviruses should be considered as a potential human pathogen (156).

Poxvirus Infection

Sealpox is caused by a parapox virus, of the family Poxviridae, and manifests as nodular, proliferative lesions of the skin in seals. Transmission to humans, leading to nodular lesions on the hands, has been reported through handling of infected seals (114). Another parapox virus is also responsible for orf (contagious ecthyma), which causes lesions in humans identical to seal pox. The parapoxvirus has been detected in proliferative lesions of muskoxen in the Canadian and Norwegian Arctic (157,158) and in mountain goats (*Oreamnos americanus*), Dall's sheep (*Ovis dalli*), muskoxen, caribou, and Sitka black-tailed deer (*Odocoileus hemionus sitkensis*) in Alaska (159), with all species having the potential to infect humans through direct contact.

Rabies

In the Western Hemisphere, rabies is maintained in wild carnivores and bats. Across Arctic and boreal regions, red and Arctic foxes, wolves, and bats (*Chiroptera*) (boreal only) pose the greatest

risk for rabies transmission to domestic dogs and humans via bites, with the disease considered endemic in Arctic foxes (81,160–162,164,166). Other wild species commonly hunted in boreal and/or Arctic regions that have tested positive for rabies include caribou, beaver, black and polar bears, lynx, and a wolverine (166,167). Rodents and hares have tested positive but are most likely dead-end hosts. Arctic foxes may very occasionally spread infection to marine mammals (168). Rabies affects the central nervous system causing general weakness or discomfort, fever, headache, prickling sensation at the site of the bite, anxiety, confusion, agitation, delirium, hallucinations, hydrophobia, and insomnia. The disease in humans is almost always fatal if untreated. Many Indigenous communities in the boreal and Arctic are familiar with signs of rabies in wildlife and domestic dogs, and some report killing affected animals immediately (169). Of note, is that the virus reportedly survives well in frozen carcasses and has caused infection in dogs fed frozen fox carcasses (146,170). Avoidance of wild and domestic animals demonstrating abnormal, suspect behavior is advised, and vaccination of domestic dogs against rabies is strongly recommended, as is euthanasia, testing of animals that bite people, and post exposure prophylaxis if indicated (166). Rabies vaccination is recommended for anyone working with a known rabies reservoir.

Hepatitis E

Whilst there is currently no confirmed transmission of Hepatitis E virus (HEV) from wildlife to humans in the Arctic and boreal regions, one study found serological evidence of HEV infection in 3% of the observed Canadian Inuit population (171). Transmission to humans can occur via undercooked meat (173). The virus has been identified in free-ranging deer in boreal Canada (172), and with caribou being closely related to deer and commonly consumed in northern communities, further studies are warranted.

Fungal Diseases

Transmission of fungal zoonoses from wildlife to humans has not been reported in Indigenous Arctic or boreal communities as yet. In general there is a dearth of fungal pathogen data for fish (174). However, because fungal zoonoses do exist in more southern regions, climate changes might bring shifts in the interactions between pathogens, hosts, and the environment. Consequently, future infections cannot be ruled out.

Prion Diseases

Chronic Wasting Disease

Chronic Wasting Disease (CWD) is an always-fatal nervous system disease of cervids that has been found in moose, deer species and elk in North America, and wild reindeer in Norway (175,176). Although it has yet to be found in North American caribou, most jurisdictions are preparing for this eventuality. Chronic Wasting Disease is a transmissible spongiform encephalopathy (TSE) caused by a naturally occurring infectious protein, called a prion, that

becomes misfolded, builds up in and damages an animal's nervous system. It can take as long as two years before an animal begins to show clinical signs of the disease (175).

There is currently no documentation of CWD transmission to humans, either through contact with infected animals or by eating meat of infected animals. However, other TSEs have spread from animals to humans via consumption of infected meat products (bovine spongiform encephalopathy or Mad Cow Disease), with a very prolonged incubation period in people; and experimental research has shown potential for CWD transmission to non-human primates, and ability of CWD to convert human prion protein to a misfolded state (366). CWD is an emerging disease and more longitudinal research is warranted; thus, public health officials advise hunters not to consume meat from animals that appear emaciated or sick with clinical signs compatible with CWD, nor to consume meat from animals that test positive for CWD. In addition, hunters are recommended to take precautions when field dressing and processing caribou and moose.

A further health consideration of CWD is that mortality in wild cervids could affect food security of subsistence-oriented communities in Arctic regions. Communities in the Northwest Territories and Nunavut with less access to employment, income, and food stores would be constrained in their ability to obtain an adequate diet in the event of scarcity of caribou meat (123).
